# Synthesis and In Vitro Evaluation of Novel Liver X Receptor Agonists Based on Naphthoquinone Derivatives

**DOI:** 10.3390/molecules24234316

**Published:** 2019-11-26

**Authors:** Tatsuma Nishioka, Kaori Endo-Umeda, Yuki Ito, Akane Shimoda, Atsuko Takeuchi, Chisato Tode, Yoshihisa Hirota, Naomi Osakabe, Makoto Makishima, Yoshitomo Suhara

**Affiliations:** 1 Laboratory of Organic Synthesis and Medicinal Chemistry, Department of Bioscience and Engineering, College of Systems Engineering and Science, Shibaura Institute of Technology, 307 Fukasaku, Minuma-ku, Saitama 337-8570, Japan; mf15065@shibaura-it.ac.jp (T.N.); mf17010@shibaura-it.ac.jp (Y.I.); mf17036@shibaura-it.ac.jp (A.S.); 2 Division of Biochemistry, Department of Biomedical Sciences, Nihon University School of Medicine, 30-1 Oyaguchi-kamicho, Itabashi-ku, Tokyo 173-8610, Japan; umeda.kaori@nihon-u.ac.jp (K.E.-U.); makishima.makoto@nihon-u.ac.jp (M.M.); 3 Instrumental Analysis Center, Kobe Pharmaceutical University, 4-19-1 Motoyamakita-machi, Higashinada-ku, Kobe 658-8558, Japan; takeuchi@kobepharma-u.ac.jp (A.T.); c-tode@kobepharma-u.ac.jp (C.T.); 4 Laboratory of Biochemistry, Department of Bioscience and Engineering, College of Systems Engineering and Science, Shibaura Institute of Technology, 307 Fukasaku, Minuma-ku, Saitama 337-8570, Japan; hirotay@sic.shibaura-it.ac.jp; 5 Bio-Intelligence for well-being Association, 307 Fukasaku, Minuma-ku, Saitama 337-8570, Japan; nao-osa@sic.shibaura-it.ac.jp; 6 Food and Nutrition Laboratory, Department of Bioscience and Engineering, College of Systems Engineering and Science, Shibaura Institute of Technology, 307 Fukasaku, Minuma-ku, Saitama 337-8570, Japan

**Keywords:** liver X receptor (LXR), naphthoquinone, α-selective, agonist, transcriptional activity

## Abstract

We aimed to synthesize novel liver X receptor (LXR) agonists with potent agonist activity and subtype selectivity. Our synthetic scheme started with naphthoquinone derivatives, such as menadione and 2,3-dichloro-1,4-naphthoquinone. We introduced different substituents into the naphthoquinone structures, including aniline, piperidine, pyrrolidine, and morpholine, in one or two steps, and thus, we produced 14 target compounds. All 14 synthetic ligands were tested to determine whether they mediated LXR-mediated transcriptional activity. We investigated the transcriptional activity of each compound with two types of receptors, LXRα and LXRβ. Among all 14 compounds, two showed weak LXRβ-agonist activity, and two others exhibited potent LXRα-agonist activity. We also performed docking studies to obtain a better understanding of the modes of compound binding to LXR at the atomic level. In conclusion, we successfully synthesized naphthoquinone derivatives that act as LXRα/β agonists and selective LXRα agonists.

## 1. Introduction

Liver X receptors (LXRs) are members of the nuclear receptor superfamily [1]. LXRs include two subtypes, LXRα and LXRβ, which have different tissue distributions. LXRα is highly expressed in liver, intestine, adipose tissue, and macrophages; in contrast, LXRβ is expressed ubiquitously in organs and tissues. These receptors are ligand-activated transcription factors involved in regulating cholesterol and lipid metabolism. An LXR agonist might be useful in preventing and treating atherosclerosis, because an agonist could promote the production of high density lipoprotein (HDL), which activates the cholesterol reverse transport system by elevating expression of the ATP-binding cassette transporter A1 and G1 genes [2,3]. Additionally, LXR agonists were shown to increase apolipoprotein E expression and decrease amyloid β protein accumulation thought to cause Alzheimer’s disease [4,5]. Thus, LXR agonists have both desirable and undesirable pharmacological effects, but ligands that act specially on LXRα or LXRβ may be applicable as therapeutic agents. A recent study reported that some LXRα/β selective agonists have been found in natural products or had been chemically synthesized [6,7,8,9,10,11]. LXRα regulates human cholesteryl ester transfer protein expression, which plays an important role in reverse cholesterol transport, both in vitro and in transgenic mice. On the other hand, LXRβ-selective agonists may be useful as therapeutic agents for arteriosclerosis and Alzheimer’s disease [7,10,11].

**Figure 1:**
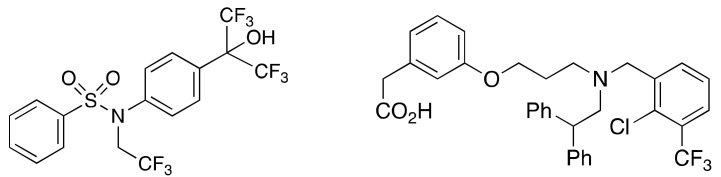
Chemical structures of T0901317 (***left***) and GW3965 (***right***).

Many previous studies have described synthetic LXR agonists, including T0901317 and GW3965, which are known potent LXR agonists (Figure 1). However, most LXR agonists have not shown subtype selectivity [6]. As aforementioned, an LXR agonist with selectivity for an LXR subtype could be a useful biologically active compound. Therefore, we aimed to synthesize novel LXR agonists that possessed potent agonistic activity as well as subtype selectivity.

We focused on synthetic compounds which have naphthoquinone moiety since they showed various biological activities. For example, some naphthoquinone derivatives have previously been described as antitumor agents [12,13]. Recently, several types of LXR agonists that bore a naphthoquinone moiety were reported [14,15]. We employed the chemical structure of those known derivatives as active motifs for our compounds. We anticipated that synthetic compounds bearing the naphthoquinone skeleton could apply for LXR agonists and the selectivity for LXRα and LXRβ would be controlled by substituents. Our design included the introduction of a nitrogen atom or a sulfur atom into the molecule, with the expectation that it would improve the interaction between the compounds and the receptor proteins. We also synthesized compounds with either a fluorine or trifluoromethyl group which is an electron-withdrawing functional group, like that incorporated into T0901317, in addition to piperidine, pyrrolidine, morpholine, aniline, and phenylsulfide moieties. At the same time, we also synthesized compounds with a methyl group, which is an electron-donating functional group, as comparators. We demonstrated that these newly synthesized LXR agonists displayed potent agonist activity, compared to known LXR agonists.

## 2. Results

### 2.1. Synthesis of LXR Ligands

**Scheme 1:**
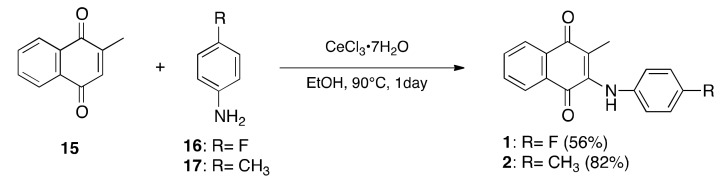
One-step synthesis of candidate liver X receptor (LXR) agonists, **1** and **2**, based on menadione (**15**).

**Scheme 2:**
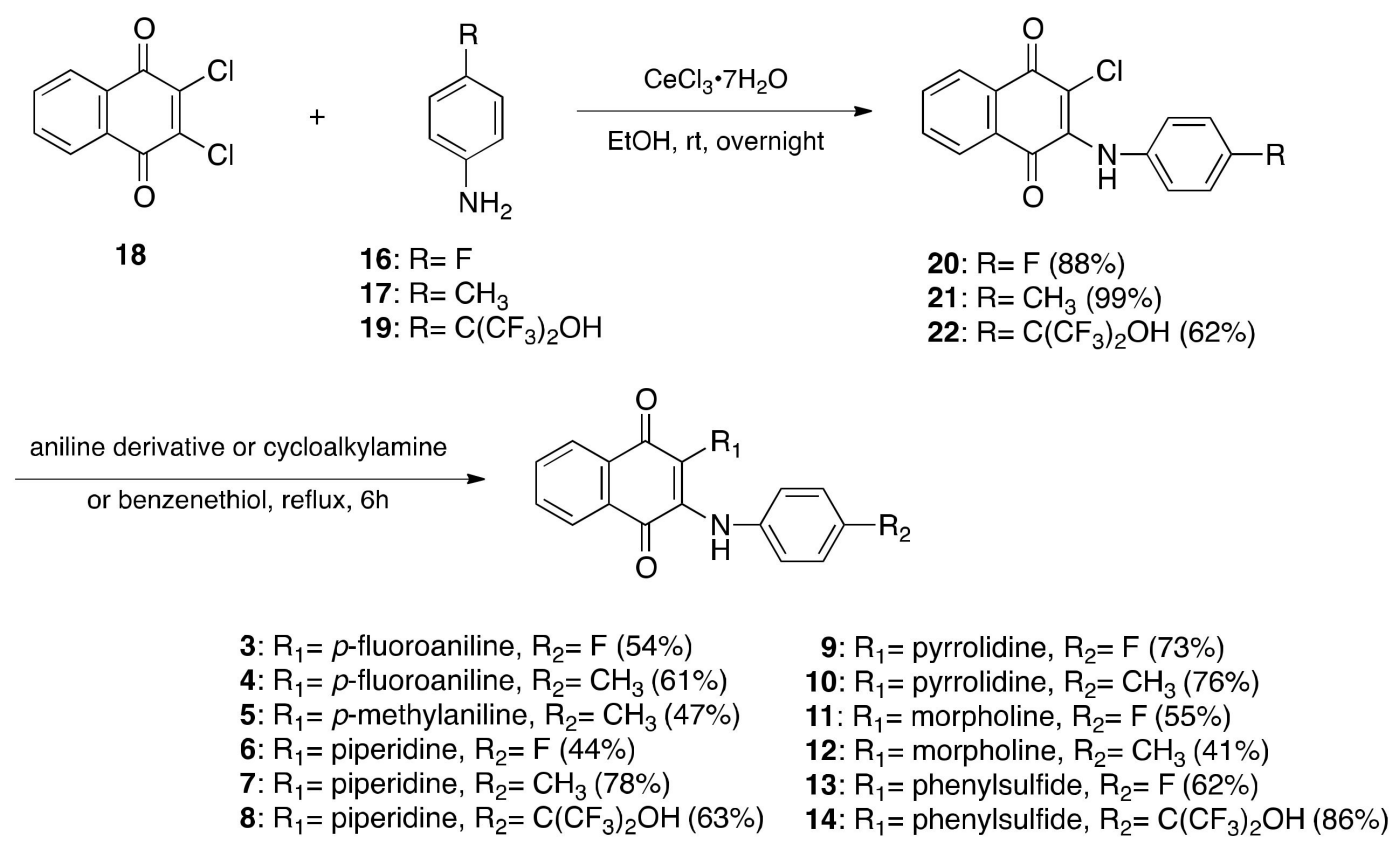
Two-step synthesis of candidate LXR agonists, **3**–**14**, based on 2,3-dichloro-1,4-naphthoquinone (**18**).

We synthesized the compounds according to the methods shown in Scheme 1 and Scheme 2. Compounds **1** and **2** were obtained in high yield, with menadione (**15**) as a starting material (Scheme 1). Briefly, we introduced an aniline derivative in the presence of cerium (III) chloride heptahydrate, according to a previously reported method [12,16,17,18]. On the other hand, compounds **3**–**14** were obtained in a 2-step synthesis method (Scheme 2). In the first step, one molecule of the aniline derivative was introduced into 2,3-dichloro-1,4-naphthoquinone (**18**) in the presence of cerium (III) chloride heptahydrate, in aqueous conditions, to produce the monochloro derivatives, **20**–**22**, in a 62–99% yield. The intermediates, **20** and **21**, were described previously [19]. In the second step, we reacted the intermediates with a nucleophilic reagent, such as excess cycloalkylamine or benzenethiol, which led to the desired compounds: **3**–**5,** with aniline derivatives; **6**–**8** with piperidine; **9**–**10** with pyrrolidine; **11**–**12** with morpholine; and **13**–**14** with phenylsulfide, in 41–86% yields. Thus, we prepared 14 different compounds that were candidate LXR agonists (The data refer to Supplementary Materials).

### 2.2. Transcriptional Activity of LXR Ligand

**Figure 2:**
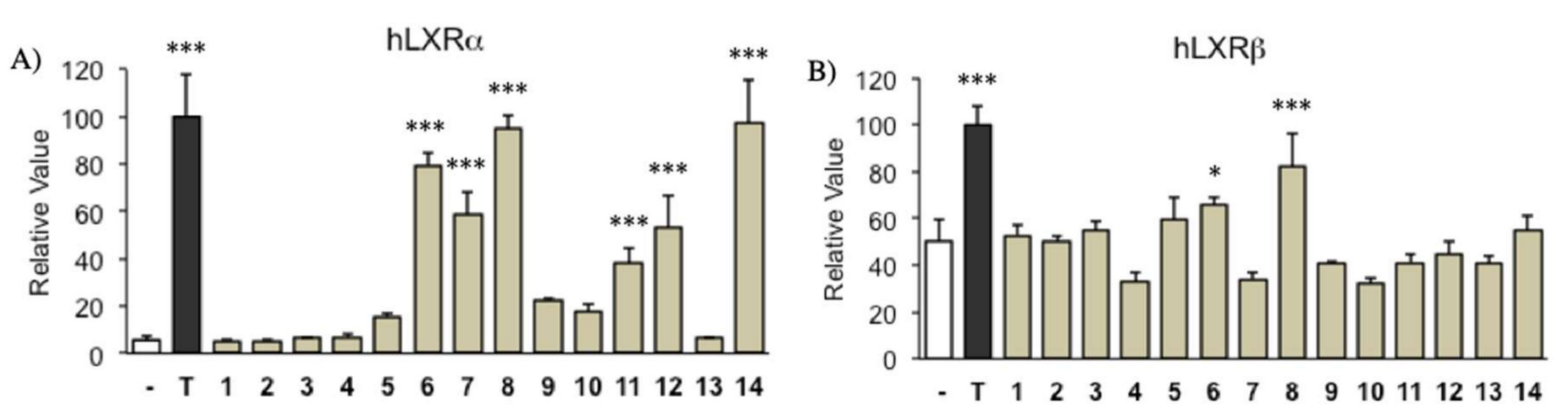
Human LXRα/β reporter gene assays in human embryonic kidney (HEK)293 cells. Treatment with compounds **1**–**14** induced reporter activity through activation of (**A**) human LXRα and (**B**) human LXRβ. T: T0901317: positive control (assigned 100%). HEK293 cells were treated with 3.0 × 10^−6^ M of each compound or 1.0 × 10^−7^ M T0901317. Negative control cells (-) were treated with DMSO alone. Data are the means of three independent experiments; error bars indicate the SD. Significant differences from the control group (-) are indicated as follows: *** *p* > 0.001, * *p* > 0.05 (Dunnett’s *t*-test).

To evaluate the agonist activity of the synthesized compounds, we tested whether they induced transcriptional activity mediated by LXRα and LXRβ with a reporter gene assay. We used T0901317 (1.0 × 10^−7^ M) as a positive control for this assay. Briefly, HEK293 cells were plated at a density that corresponded to 70–80% confluence (1 × 10^4^ cells per each well) in a 96-well plate, 24 h prior to transfection. Then, cells were co-transfected with an expression plasmid that carried one of two nuclear receptors, under the control of the ctyomegalovirus promoter (pCMX-hLXRα or pCMX-hLXRβ), a reporter plasmid (rCYP7A-DR4 × 3-tk-LUC), and a CMX-β-galactosidase vector, as an indicator of expression efficiency. Transfections were performed according to the calcium phosphate co-precipitation method. After 24 h, transfected cells were treated with test compounds (3.0 × 10^−6^ M) or dimethyl sulfoxide (DMSO) for 16 h. Treated cells were assayed for luciferase reporter activity in a luminometer. The luciferase activity measured in each sample was normalized with respect to the level of β-galactosidase activity (Figure 2) [6].

Our compounds did not show the potency of T0901317; however, the substituents on the compounds affected the transcriptional activities of LXRα and LXRβ. Significant LXRα activities, compared to control, were observed with compounds **6**, **7**, **8**, **11**, **12**, and **14**. In particular, **8** and **14**, which carried the trifluoromethyl group, exhibited the most potent induction of LXRα activity, among all our compounds. However, compounds **1**, **2**, **3**, **4**, **5**, **9**, **10**, and **13** showed no significant difference with induction of LXRα activity (Figure 2A). On the other hand, weak inductions of LXRβ activity were observed only with compounds **6** and **8**. Other compounds had no significant difference compared to control group (Figure 2B). Based on these findings, compounds **6** and **8** had agonist effects on both LXRα and LXRβ. Furthermore, from the comparison between LXRα and LXRβ, the activities of compounds **7**, **11**, **12**, and **14** suggested that these agonists were candidates with selective LXRα binding. In particular, **14** showed the highest LXRαselective activity in this assay.

## 3. Discussion

**Figure 3:**
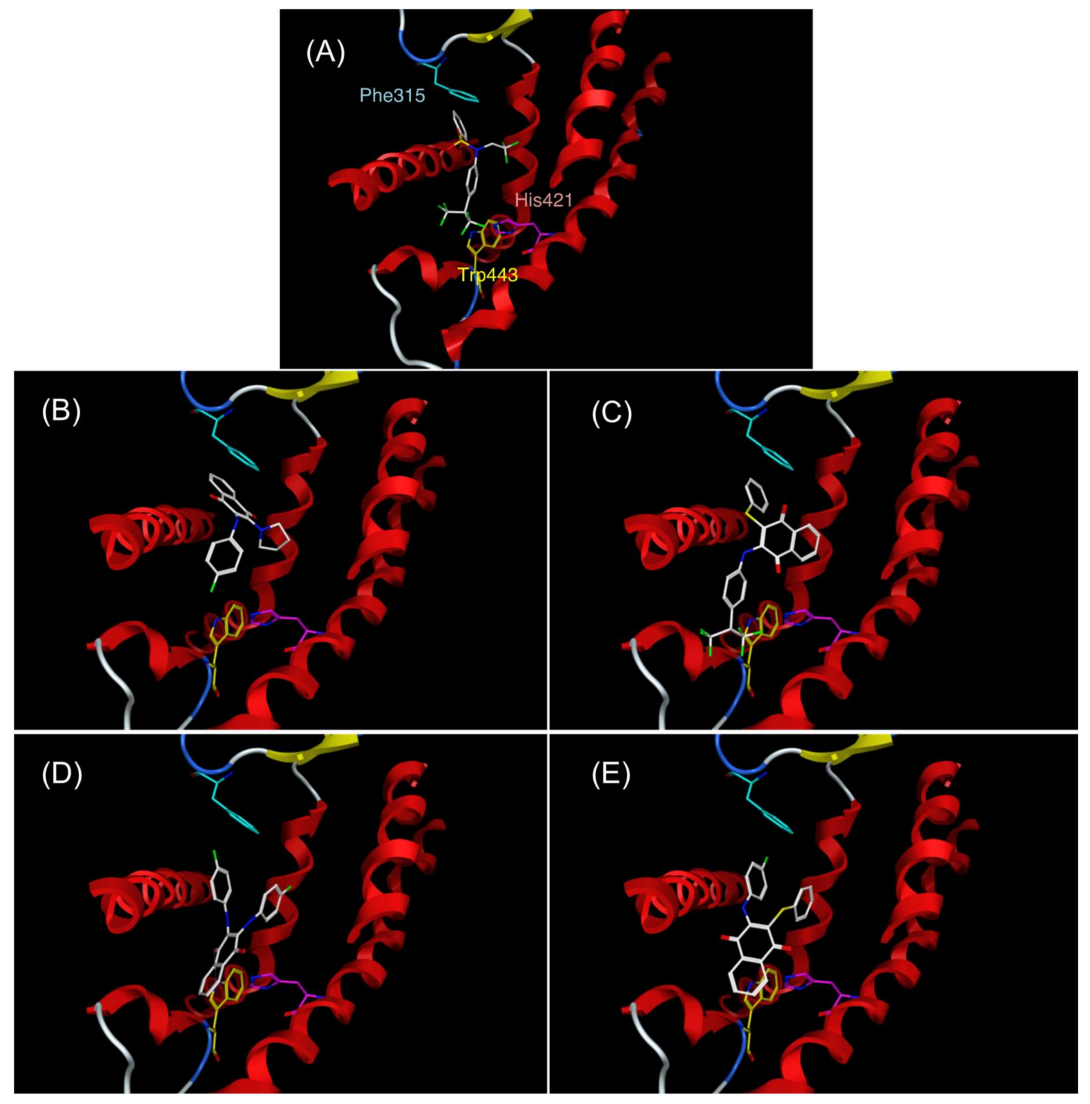
Simulations of test compounds docking to LXRα (PDB ID: 1UHL). The test compounds (*center, white*) are shown interacting with amino acid residues of LXRα (*red*). (**A**) T0901317 originally contained in 1UHL; (**B**) compound **8** and (**C**) compound **14** exhibited the most potent activity among all 14 test compounds. Compounds **8** and **14** exhibited π–π stacking interactions with Phe315 (*sky blue*), and those (CF_3_)_2_OH moiety faced to His421 (*purple*) and Trp443 (*yellow*), similar to T0901317; (**D**) Compound **3** and (**E**) compound **13** showed no agonist activity.

To better understand the binding modes of compounds to LXRs at the atomic level (based on the results shown in Figure 2), we performed molecular docking studies on compounds with LXRα (PDB ID: 1UHL [20]) using the docking program of the MOE suite (see Computational Details) as shown in Figure 3. We calculated the binding affinity by replacing T-0901317, originally bound to LXRα, with compounds using the “dock” mode. We selected compounds **8** and **14**, because they had the highest agonistic activity for LXRα, and **3** and **13** randomly picked up from **1**, **2**, **3**, **4**, **5**, **9**, **10**, and **13**, because they showed no agonist activity for LXRα. When compounds **8** and **14** were in their most stable binding conformations (it means the lowest binding energy was exhibited), their aromatic rings faced to the Phe315 (sky blue) of LXRα, which facilitated the formation of a π-π stacking interaction (Figure 3B,C). On the other hand, when compounds **3** and **13** were in their most stable binding conformations, they faced the opposite direction, and the aromatic rings did not interact with Phe315 (Figure 3D,E). Previous studies on LXR also suggested that residues His435 and Trp457 (PDB ID: 5HJP) functioned as an activation switch, and the interaction between the ligand and His435 was particularly important [7]. Therefore, we performed docking simulations to analyze interactions with His421 and Trp443 (corresponding to His435 and Trp457 in 5HJP, respectively) in the docking simulation with 1UHL. In the most stable structure of **8** and **14** bound to LXRα, we obtained a conformation similar to T0901317, with the (CF_3_)_2_OH moiety facing His421 (purple) and Trp443 (yellow) (Figure 3A–C). These similarities were suggested to show agonistic activity. However, the docking simulations could not clarify the differential effects of these compounds on LXRα and LXRβ. This observation was consistent with results reported in a previous study. Those authors speculated that ligand selectivity for LXRα vs. LXRβ arises from different post-binding conformational changes or differential coactivator recruitment, rather than from different binding preferences [6].

## 4. Materials and Methods

### 4.1. Synthetic Method of Compounds

#### 4.1.1. General Experimental Procedures

^1^H and ^13^C-NMR spectra were recorded at 400 and 100 MHz in CDCl_3_ or CD_3_OD, with tetramethylsilane used as an internal standard. High-resolution mass spectra were measured in the time of flight (TOF) mass mode.

#### 4.1.2. Preparation of 2-[(4-Fluorophenyl)amino]-3-methylnaphthalene-1,4-dione (**1**)

Menadione (**15**) (172 mg, 1.00 mol), 4-fluoroaniline (**16**) (194 µL, 2.00 mmol), and CeCl_4_•7H_2_O (19 mg, 50.0 µmol) were dissolved in 15 mL of ethanol, and then heated to reflux at 90 °C overnight. The reaction mixture was poured into ice-water and extracted with CH_2_Cl_2_ (100 mL × 3). The combined organic layer was washed with water (100 mL) and brine (100 mL) and dried over MgSO_4_ to concentrate the product. The residue was purified with silica gel column chromatography (*n*-hexane/AcOEt = 6:1) to afford compound **1** (163 mg, 58%) as an orange powder: ^1^H-NMR (500 MHz, CDCl_3_) δ1.72 (3H, s), 6.97–7.05 (4H, m), 7.33 (1H, s), 7.64–7.72 (2H, m), 8.06–8.12 (2H, m); ^13^C-NMR (125 MHz, CDCl_3_) δ13.6, 115.5, 115.7, 118.2, 124.2, 124.3, 126.2, 126.3, 132.4, 134.4, 158.3, 160.7, 182.4, 184.4; HRMS ([M + H]^+^) *m/z* was calculated for C_17_H_12_FNO_2_ 282.0930; Found: 282.0925.

#### 4.1.3. Preparation of 2-Methyl-3-[(4-methylphenyl)amino]naphthalene-1,4-dione (**2**)

Similar to the synthesis of **1** from **15** and 4-fluoroaniline (**16**), the crude product 2, which was obtained from **15** (344 mg, 2.00 mmol), *p*-toluidine (**17**) (430 mg, 4.00 mmol), and CeCl_4_•7H_2_O (38 mg, 100 μmol) in ethanol (30 mL), was purified with silica gel column chromatography (*n*-hexane/AcOEt = 6:1), which gave compound **2** (229 mg, 82%) as a purple powder: ^1^H-NMR (400 MHz, CDCl_3_) δ 1.73 (3H, s), 2.37 (3H, s), 6.88–6.90 (2H, d), 7.11–7.13 (2H, d), 7.37 (1H, s), 7.60–7.72 (2H, m), 8.04–8.11 (2H, m); ^13^C-NMR (100 MHz, CDCl_3_) δ 13.7, 16.5, 20.8, 117.8, 122.6, 126.0, 126.1, 126.2, 126.5, 129.3, 132.2, 133.5, 133.6, 134.0, 134.3, 135.6, 137.1, 182.6, 184.4, 184.9, 185.5; HRMS ([M + H]^+^) *m/z* calculated for C_18_H_16_NO_2_ 278.1181; Found: 278.1177.

#### 4.1.4. Preparation of 2-chloro-3-[(4-fluorophenyl)amino]naphthalene-1,4-dione (**20**)

2,3-Dichloro-1,4-naphthoquinone (**18**) (909 mg, 4.00 mmol), **16** (767 µL, 8.00 mmol), and CeCl_4_ (75 mg, 200 µmol) were dissolved in 15 mL of ethanol, and stirred overnight. The reaction mixture was poured into ice-water and extracted with CH_2_Cl_2_ (100 mL × 3). The combined organic layer was washed with water (100 mL) and brine (100 mL), and dried over MgSO_4_ to concentrate the product. The residue was purified with silica gel column chromatography (*n*-hexane/AcOEt = 3:1) to afford **20** (1.23 g, 88%) as a dark red powder.

#### 4.1.5. Preparation of 2-Chloro-3-[(4-methylphenyl)amino]naphthalene-1,4-dione (**21**)

Similar to the synthesis of **20** from **16** and **18**, the crude product **21**, which was obtained from **18** (681 mg, 3.00 mmol), **17** (645 mg, 6.00 mmol), and CeCl_4_•7H_2_O (57 mg, 150 μmol) in ethanol (15 mL), was purified with silica gel column chromatography (n-hexane/AcOEt = 3:1), which gave compound **21** (890 mg, 99%) as a purple powder.

#### 4.1.6. Preparation of 2-Chloro-3-{[4-(1,1,1,3,3,3-hexafluoro-2-hydroxypropan-2-yl)phenyl]amino} naphthalene-1,4-dione (**22**)

Similar to the synthesis of **20** from **16** and **18**, the crude product **22**, which was obtained from **18** (341 mg, 1.50 mmol), 2-(4-aminophenyl)-1,1,1,3,3,3-hexafluoropropan-2-ol (**19**) (441 mg, 1.70 mmol), and CeCl_4_•7H_2_O (28 mg, 75 μmol) in ethanol (15 mL), was purified with silica gel chromatography (n-hexane/AcOEt = 3:1), which gave compound **22** (416 mg, 62%) as a red powder: ^1^H-NMR (400 MHz, CD_3_OD) δ 7.19 (dt, 2H, *J* = 8.8, 2.6 Hz), 7.68 (d, 2H, *J* = 8.4 Hz), 7.79 (m, 2H), 8.11 (dd, 2H, *J* = 8.8, 1.2 Hz); ^13^C-NMR (CD_3_OD, 100 MHz): δ 115.0, 121.8, 123.3, 124.6, 126.3, 126.5, 126.8, 127.4, 130.4, 132.3, 133.0, 134.5, 140.2, 142.9, 177.8, 180.0; HRMS ([M + H]^+^) *m/z* calculated for C_19_H_11_ClF_6_NO_3_ 450.0332; Found: 450.0332.

#### 4.1.7. Preparation of 2-[(4-Fluorophenyl)amino]-3-(piperidin-1-yl)naphthalene-1,4-dione (**3**)

To a solution of compound **20** (302 mg, 1.00 mmol) in toluene (25 mL), we added **16** (144 µL, 1.50 mmol), sodium butoxide (144 mg, 1.50 mmol), palladium chloride (188 mg, 230 µmol), and 1,1′-bis(diphenylphosphino)ferrocene (128 mg, 230 µmol); the mixture was stirred at 100 °C for 6 h under Ar. The reaction mixture was poured into ice-water and extracted with CH_2_Cl_2_ (100 mL × 3). The combined organic layer was washed with water (100 mL) and brine (100 mL), and dried over MgSO_4_ to concentrate the product. The residue was purified with silica gel column chromatography (*n*-hexane/AcOEt = 4:1) to afford **3** (204 mg, 54%), as a navy-blue powder: ^1^H-NMR (400 MHz, CDCl_3_) δ 6.31 (4H, m), 6.64-6.66 (4H, m), 7.19 (2H, d), 7.69 (2H, m), 8.07–8.11 (2H, m); ^13^C-NMR (100 MHz, CDCl_3_) δ 114.1, 114.3, 121.8, 121.9, 123.5, 126.4, 131.4, 133.1, 133.1,133.7, 157.3, 159.7, 181.3; HRMS ([M + H]^+^) *m/z* calculated for C_22_H_15_N_2_O_2_F_2_ 377.1102; Found: 377.1102

#### 4.1.8. Preparation of 2-[(4-Fluorophenyl)amino]-3-(p-tolylamino)naphthalene-1,4-dione (**4**)

Similar to the synthesis of **3** from **20** and **16**, the crude product **4**, which was obtained from **21** (298 mg, 1.00 mmol), **16** (144 µL, 1.50 mmol), sodium butoxide (144 mg, 1.50 mmol), palladium chloride (188 mg, 230 µmol), and 1,1′-bis(diphenylphosphino)ferrocene (128 mg, 230 µmol) in toluene (25 mL), was purified with silica gel chromatography (*n*-hexane/AcOEt = 4:1), which gave compound **4** (229 mg, 61%) as a navy-blue solid: ^1^H-NMR (400 MHz, CDCl_3_) δ 2.18 (3H, s), 6.24–6.28 (4H, m), 6.58–6.75 (4H, m), 7.25 (2H, d), 7.64–7.66 (2H, m), 8.06–8.08 (2H, m); ^13^C-NMR (100 MHz, CDCl_3_) δ 20.8, 113.9, 114.1, 120.6, 121.6, 121.7, 126.3, 126.3, 128.0, 133.5, 133.6, 157.2, 159.6, 181.3, 181.5; HRMS ([M + Na]^+^) *m/z* calculated for C_23_H_17_N_2_O_2_FNa 395.1172; Found: 395.1172.

#### 4.1.9. Preparation of 2,3-Bis[(4-methylphenyl)amino]naphthalene-1,4-dione (**5**)

Similar to the synthesis of **3** from **20** and **16**, the crude product **5**, which was obtained from **21** (298 mg, 1.00 mmol), **17** (161 mg, 1.50 mmol), sodium butoxide (144 mg, 1.50 mmol), palladium chloride (188 mg, 230 µmol), and 1,1′-bis(diphenylphosphino)ferrocene (128 mg, 230 µmol) in toluene (25 mL), was purified with silica gel chromatography (n-hexane/AcOEt = 4:1), which gave compound **5** (175 mg, 47%) as a navy-blue powder: ^1^H-NMR (400 MHz, CDCl_3_) δ 2.17 (6H, s), 6.23–6.25 (4H, m), 6.69–6.71 (4H, m), 7.14–7.25 (2H, d), 7.62–7.65 (2H, m), 8.05–8.08 (2H, m); ^13^C-NMR (100 MHz, CDCl_3_): δ 20.7, 120.3, 123.6, 126.1, 127.8, 131.3, 131.5, 133.3, 134.8, 181.3; HRMS ([M + Na]^+^) *m/z* calculated for C_24_H_20_N_2_O_2_Na 391.1422; Found: 391.1422.

#### 4.1.10. Preparation of 2[(4-Fluorophenyl)amino]-3-(piperidin-1-yl)naphthalene-1,4-dione (**6**)

Compound **20** (302 mg, 1.00 mol) was dissolved in piperidine (5 mL), and heated to reflux at 125 °C for 1 day. After cooling to room temperature, the mixture was poured into water and extracted with ethyl acetate (100 mL × 3). The combined organic layer was washed with water (100 mL) and brine (100 mL) and dried over MgSO_4_ to concentrate the product. The residue was purified with silica gel column chromatography (n-hexane/AcOEt = 5:1) to afford **6** (154 mg, 44%) as a navy-blue powder. ^1^H-NMR (400 MHz, CDCl_3_) δ 1.21–1.59 (6H, m), 3.06–3.07 (4H, m), 6.78–6.83 (2H, m), 6.91–6.96 (2H, m), 7.10 (1H, br s), 7.55–7.65 (2H, m), 7.96–8.00 (2H, m); ^13^C-NMR (100 MHz, CDCl_3_): δ 24.2, 26.1, 49.9, 114.8, 115.0, 121.7, 121.8, 125.6, 126.4, 130.7, 132.7, 133.67, 133.71, 157.4, 159.8, 182.0, 182.3; HRMS (M + H^+^) *m/z* calculated for C_21_H_20_O_2_N_2_F 351.1509. Found: 351.1508.

#### 4.1.11. Preparation of 2-(Piperidin-1-yl)-3-[(4-methylphenyl)amino]naphthalene-1,4-dione (**7**)

Similar to the synthesis of **6** from **20** and piperidine, the crude product 7, which was obtained from **21** (298 mg, 1.00 mmol) and piperidine (5 mL), was purified with silica gel chromatography (n-hexane/AcOEt = 5:1), which gave compound **7** (271 mg, 76%) as a navy-blue powder: ^1^H-NMR (400 MHz, CDCl_3_) δ 1.26–1.38 (6H, m), 2.32 (3H, s), 3.08–3.11 (4H, m), 6.76–6.78 (2H, m), 7.04–7.06 (2H, m), 7.11(1H, br s), 7.58–7.63 (2H, m), 7.97–8.01 (2H, m); ^13^C-NMR (100 MHz, CDCl_3_): δ 21.0, 24.3, 26.1, 49.8, 120.3, 125.6, 126.3, 128.8, 130.8, 131.9, 132.6, 132.9, 133.6, 133.7, 137.3, 182.0, 182.4. HRMS (M + H^+^) *m/z* calculated for C_22_H_23_O_2_N_2_ 347.1760. Found: 351.1750.

#### 4.1.12. Preparation of 2-{[4-(1,1,1,3,3,3-Hexafluoro-2-hydroxypropan-2-yl)phenyl]amino}-3-(piperidin-1-yl)naphthalene- 1,4-dione (**8**)

Similar to the synthesis of **6** from **20** and piperidine, the crude product **8**, which was obtained from **22** (225 mg, 0.50 mmol) and piperidine (5 mL), was purified with silica gel chromatography (n-hexane/AcOEt = 5:1), which gave compound **8** (157 mg, 63%) as a yellow solid: ^1^H-NMR (400 MHz, CDCl_3_) δ 1.26–1.35 (7H, m), 3.13–3.16 (4H, m), 6.88–6.90 (2H, m), 7.16 (1H, br s), 7.54–7.72 (4H, m), 8.01–8.03 (2H, m); ^13^C-NMR (100 MHz, CDCl_3_): δ 24.1, 25.9, 49.9, 119.6, 122.4, 125.7, 126.6, 126.7, 128.8, 130.7, 132.7, 133.0, 133.6, 135.4, 141.4, 182.2, 182.3; HRMS (M + H^+^) *m/z* calculated for C_24_H_21_O_2_N_2_F_6_ 499.1456. Found: 499.1456.

#### 4.1.13. Preparation of 2-[(4-Fluorophenyl)amino]-3-(pyrrolidin-1-yl)naphthalene-1,4-dione (**9**)

Compound **20** (302 mg, 1.00 mol) was dissolved in pyrrolidine (5 mL), and heated to reflux at 107 °C for 6 h. After cooling to room temperature, the mixture was poured into water and extracted with ethyl acetate (100 mL × 3). The combined organic layer was washed with water (100 mL) and brine (100 mL) and dried over MgSO_4_ to concentrate the product. The residue was purified with silica gel column chromatography (n-hexane/AcOEt = 5:1) to afford **9** (248 mg, 73%) as a navy-blue powder. ^1^H-NMR (400 MHz, CDCl_3_) δ 1.62-1.63 (4H, m), 3.44-3.48 (4H, m), 6.58 (2H, m), 6.88–6.90 (3H, m), 7.60 (2H, m), 7.96–8.01 (2H, m); ^13^C-NMR (100 MHz, CDCl_3_): δ 25.4, 51.1, 115.1, 115.3, 117.2, 117.3, 123.4, 125.5, 126.1, 131.5, 132.7, 133.3, 156.1, 158.4, 180.5, 183.3; HRMS ([M + H]^+^) *m/z* calculated for C_20_H_18_N_2_O_2_F 377.1352; Found: 337.1351.

#### 4.1.14. Preparation of 2-(Pyrrolidin-1-yl)-3-[(4-methylphenyl)amino]naphthalene-1,4-dione (**10**)

Similar to the synthesis of **9** from **20** and pyrrolidine, the crude product 10, which was obtained from **21** (298 mg, 1.00 mmol) and pyrrolidine (5 mL), was purified with silica gel chromatography (n-hexane/AcOEt = 5:1), which gave compound **10** (255 mg, 76%) as a navy-blue powder: ^1^H-NMR (400 MHz, CDCl_3_) δ 1.61–1.63 (4H, m), 2.17 (3H, s), 3.46–3.49 (4H, m), 6.52–6.55 (2H, m), 6.81 (1H, s), 6.98–7.00 (2H, m), 7.59–7.60 (2H, m), 7.97–8.00 (2H, m); ^13^C-NMR (100 MHz, CDCl_3_): δ 20.6, 25.4, 50.9, 116.1, 123.5, 125.3, 125.9, 129.1, 129.4, 131.5, 131.9, 132.5, 133.1, 136.7, 139.9, 180.4, 183.3; HRMS ([M + H]^+^) *m/z* calculated for C_21_H_21_N_2_O_2_ 333.1603; Found: 333.1597.

#### 4.1.15. Preparation of 2-[(4-Fluorophenyl)amino]-3-morpholinonaphthalene-1,4-dione (**11**)

Compound **20** (302 mg, 1.00 mol) was dissolved in morpholine (5 mL), and heated to reflux at 140 °C for 2 days. After cooling to room temperature, the mixture was poured into water and extracted with ethyl acetate (100 mL × 3). The combined organic layer was washed with water (100 mL) and brine (100 mL) and dried over MgSO_4_ to concentrate the product. The residue was purified with silica gel column chromatography (*n*-hexane/AcOEt = 5:1) to afford **11** (196 mg, 55%) as a black powder. ^1^H-NMR (400 MHz, CDCl_3_) δ ^1^H-NMR (400 MHz, CDCl_3_): δ 3.15–3.18 (4H, m), 3.32–3.44 (4H, m), 6.87–6.90 (2H, m), 6.98–7.02 (2H, m), 7.27 (1H, br s), 7.62–7.69 (2H, m), 8.01–8.03 (2H, m); ^13^C-NMR (100 MHz, CDCl_3_): δ 48.9, 66.8, 115.0, 115.2, 122.7, 122.8, 125.8, 126.5, 130.4, 131.3, 132.8, 134.1, 157.8, 160.2, 182.0, 182.5; HRMS (M + H^+^) *m/z* calculated for C_20_H_18_O_3_N_2_F 353.1301. Found: 353.1299.

#### 4.1.16. Preparation of 2-Morpholino-3-[(4-methylphenyl)amino]naphthalene-1,4-dione (**12**)

Similar to the synthesis of **11** from **20** and morpholine, the crude product **12**, which was obtained from **21** (298 mg, 1.00 mmol) and morpholine (5 mL), was purified with silica gel chromatography (n-hexane/AcOEt = 5:1), which gave compound **12** (145 mg, 41%) as a black powder: ^1^H-NMR (400 MHz, CDCl_3_): δ 2.34 (3H, s), 3.16–3.18 (4H, m), 3.31–3.33 (4H, m), 6.81–6.83 (2H, m), 7.09–7.11 (2H, m), 7.21 (1H, s), 7.601–7.68 (2H, m), 8.00–8.03 (2H, m); ^13^C-NMR (100 MHz, CDCl_3_): δ 21.0, 48.9, 66.8, 121.5, 125.8, 126.4, 128.9, 130.5, 131.1, 132.3, 132.6, 132.9, 133.0, 134.0, 136.3, 181.9, 182.7; HRMS (M + H^+^) *m/z* calculated for C_21_H_21_O_3_N_2_ 349.1552. Found: 349.1548.

#### 4.1.17. Preparation of 2-[(4-Fluorophenyl)amino]-3-(phenylthio)naphthalene-1,4-dione (**13**)

To a stirred solution of thiophenol (45 µL, 0.32 mmol), triethylamine (45 µL, 0.32 mmol), potassium hydroxide (107 mg, 1.9 mmol), and a 0.5% SDS solution (5 mL), we added compound **20** (97 mg, 0.32 mmol) and heated to 60 °C for 14 h. After cooling to room temperature, the mixture was neutralized with 5% hydrochloric acid solution. Water was added to the mixture, and the solution was extracted with dichloromethane (100 mL × 3). The combined organic layer was dried over MgSO_4_ to concentrate the product. The residue was purified with silica gel column chromatography (n-hexane/AcOEt = 15:1) to afford **13** (75 mg, 62%) as a red-purple powder: ^1^H-NMR (400 MHz, CDCl_3_): δ 6.62–6.65 (2H, m), 6.71–6.75 (2H, m), 6.81–6.85 (2H, m), 7.00–7.10 (3H, m), 7.67–7.78 (2H, m), 7.91 (1H, br s), 8.10–8.19 (2H, m); ^13^C-NMR (100 MHz, CDCl_3_): δ 114.4, 114.6, 124.8, 125.9, 126.9, 127.2, 127.9, 128.5, 133.0, 135.0, 142.6, 158.9, 161.3, 180.8, 181.3; HRMS (M + H^+^) *m/z* calculated for C_22_H_15_O_2_NFS 376.0808. Found: 376.0808.

#### 4.1.18. Preparation of 2-{[4-(1,1,1,3,3,3-Hexafluoro-2-hydroxypropan-2-yl)phenyl]amino}-3-(phenylthio)naphthalene-1,4-dione (**14**)

Thiophenol (33 µL, 0.32 mmol), trimethylamine (45 µL, 0.32 mmol), and compound **22** (144 mg, 0.32 mmol) were dissolved in methanol (50 mL) and heated at 70 °C for 24 h. After cooling to room temperature, water was added to the mixture and the solution was extracted with dichloromethane (100 mL × 3). The combined organic layer was dried over MgSO_4_ to concentrate the product. The residue was purified with silica gel column chromatography (n-hexane/AcOEt = 10:1) to afford **14** (144 mg, 86%) as a red-brown powder: ^1^H-NMR (400 MHz, CDCl_3_): δ 3.59 (1H, br s), 6.53–6.55 (2H, m), 6.59–6.61 (2H, m), 6.90–6.94 (2H, m), 6.98–7.03 (1H, m), 7.45–7.47 (2H, d, *J* = 8.0 Hz), 7.71–7.81 (2H, m), 7.95 (1H, br s), 8.14–8.22 (2H, m); ^13^C-NMR (100 MHz, CDCl_3_): δ 115.1, 121.8, 124.5, 125.8, 126.3, 127.0, 127.2, 128.3, 128.7, 130.4, 131.2, 133.3, 135.0, 136.9, 139.6, 180.6, 181.8; HRMS (M + H^+^) *m/z* calculated for C_25_H_16_O_3_NF_6_S 524.0755. Found: 524.0756.

### 4.2. Cell Culture Conditions

Human embryonic kidney (HEK) 293 cells were cultured in DMEM containing 5% FBS and a penicillin/streptomycin mixture, at 37 °C, in a humidified atmosphere of 5% CO_2_ in air.

### 4.3. Transient Transfection Assays

HEK293 cells were plated at a density corresponding to 70–80% confluence (1 × 10^4^ cells per each well) in a 96-well plate, 24 h prior to transfection. Cells were co-transfected with 15 ng of an expression plasmid that carried one of two nuclear receptors (pCMX-hLXRα/β), 50 ng of reporter plasmid (rCYP7A-DR4×3-tk-LUC), and 10 ng of pCMX-β-galactosidase expression vector. Transfection was performed according to the calcium phosphate co-precipitation method. After 24 h, transfected cells were treated with test compounds or DMSO for 16 h. Agonist activity was measured in response to each test compound (3.0 × 10^−6^ M) or T0901317 (1.0 × 10^−7^ M). Treated cells were assayed for luciferase activity in a luminometer. The luciferase activity of each sample was normalized to the level of β-galactosidase activity. Each transfection was carried out twice in triplicate. Error bars represent SD. Statistics were performed with the Dunnett’s *t*-test.

### 4.4. Computational Details

Calculations were performed on a Dell Precision T3500 workstation. Conformational analysis of T0901317 and compounds **3**, **8**, **13**, **14** were conducted using Molecule Operating Environment (MOE, 2019.01; Chemical Computing Group Inc., 1010 Sherbrooke St. West, Suite #910, Montreal, QC, Canada) and Amber10: EHT molecular mechanics force field [21]. Docking of the minimized energy structure of the compounds into the crystal structure of LXR𝛼 in complex with T0901317 and compounds were carried out with the docking program of the MOE suite.

### 4.5. Molecular Docking Experiment

The drug-bound LXR𝛼 structure (PDB code: 1UHL, Resolution: 2.9 A) was obtained from Protein Data Bank (PDB) [20]. The cocrystalized structure was prepared using MOE 2019.01 for correcting structural issues (such as break bond, miss loop, etc.), adding hydrogen, and calculating partial charge. The 2D structure of the compounds were downloaded from the CS ChemDraw with mol file format and converted to 3D in MOE through energy minimization. MOE-Docking was used for docking simulation of the compounds and predicting the binding affinity with the LXR𝛼 protein structure. The original drug-binding pocket was chosen as the active site for docking. Site Finder in MOE was also used to identify the potential binding pockets and analyze the conserved pocket residues. Classical triangle matching was chosen as placement method, and the number of placement poses was set to 100. The output docking poses were evaluated by the London dG score and top 30 poses were chosen. Then, the rigid receptor method was employed in the refinement step. The number of the final output docking poses was set to 20, followed by minimizing using Amber10: EHT force field in MOE. The GBVI/WSA dG score was used to estimate free energy of binding of the compounds with the LXR𝛼. The binding mode was analyzed in MOE after the refinement minimization [22,23].

## 5. Conclusions

In summary, we prepared a series of compounds based on naphthoquinone derivatives that displayed selective binding to LXRα. In future studies, we will examine the skeletons of these compounds or the structural modifications that afford the best selectivity for LXRα vs. LXRβ.

## Supplementary Materials

The following are available online. Figure S1: ^1^H and ^13^C NMR chart of **1**–**14**.
