# Supplementary material for: Synthesis and In Vitro Evaluation of Novel Liver X Receptor Agonists Based on Naphthoquinone Derivatives
Source: Molecules. 2019 Nov 26;24(23):4316. doi: 10.3390/molecules24234316 (PMC6930623; doi:10.3390/molecules24234316)
Supplement: Supplementary file 1 [file molecules-24-04316-s001.pdf]

Supplementary Material

Synthesis and in vitro evaluation of novel liver X receptor agonists based on naphthoquinone derivatives

Tatsuma Nishioka[1,†], Kaori Endo-Umeda[2,†], Yuki Ito[1], Akane Shimoda[1], Atsuko Takeuchi[3], Chisato Tode[3], Yoshihisa Hirota[4,6], Naomi Osakabe[5,6], Makoto Makishima[2], and Yoshitomo Suhara[1,6]*

[1]*Laboratory of Organic Synthesis and Medicinal Chemistry, Department of Bioscience and Engineering, College of Systems Engineering and Science, Shibaura Institute of Technology, 307 Fukasaku, Minuma-ku, Saitama 337-8570, Japan*
[2]*Division of Biochemistry, Department of Biomedical Sciences, Nihon University School of Medicine*
[3]*Intrumental Analysis Center, Kobe Pharmaceutical University, 4-19-1 Motoyamakita-machi, Higashinada-ku, Kobe 658-8558, Japan*
[4]*Laboratory of Biochemistry, Department of Bioscience and Engineering, College of Systems Engineering and Science, Shibaura Institute of Technology, 307 Fukasaku, Minuma-ku, Saitama 337-8570, Japan*
[5]*Food and Nutrition Laboratory, Department of Bioscience and Engineering, College of Systems Engineering and Science, Shibaura Institute of Technology, 307 Fukasaku, Minuma-ku, Saitama 337-8570, Japan*
[6]*Bio-Intelligence for well-being Association, 307 Fukasaku, Minuma-ku, Saitama 337-8570, Japan*

* Correspondence: suhara@shibaura-it.ac.jp; Tel. +81-48-720-6043

† Equally contributed.

Figure S1

I. ^{1}H and ^{13}C NMR chart of **1 – 14**

^{1}H NMR chart of compound **1**

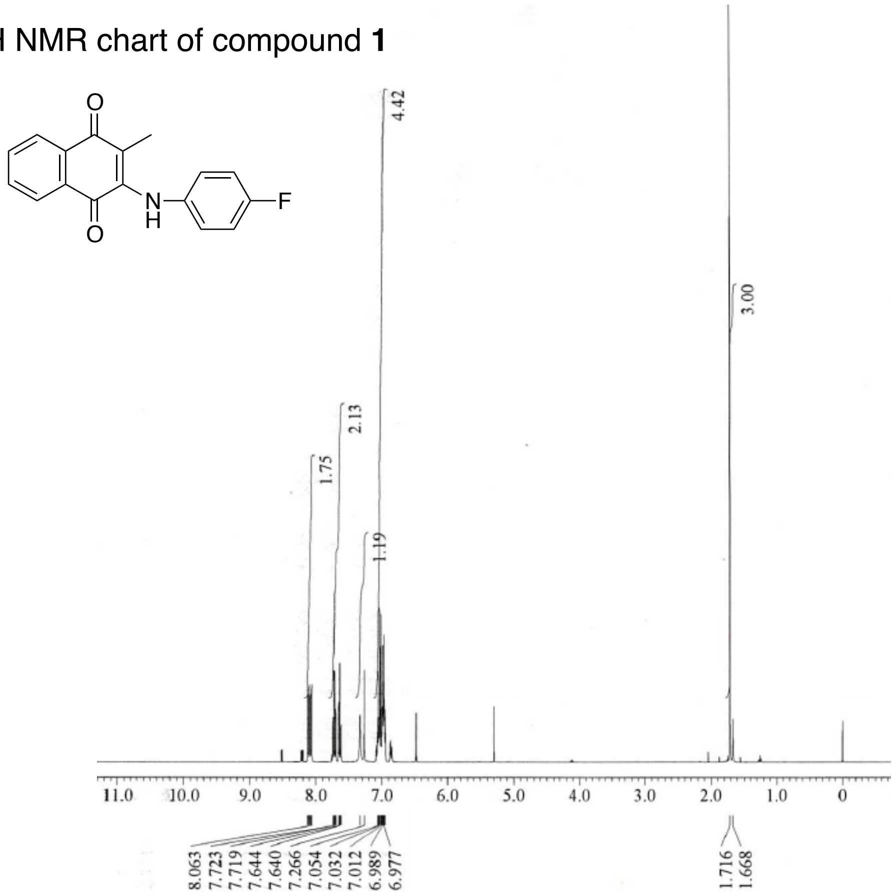

^{13}C NMR chart of compound **1**

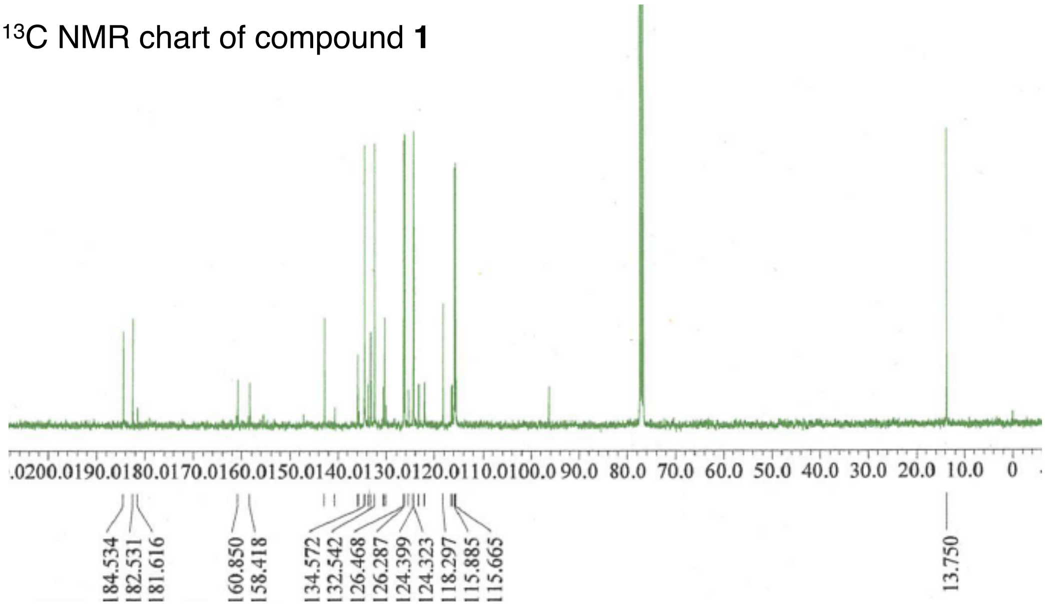

^{1}H NMR chart of compound **2**

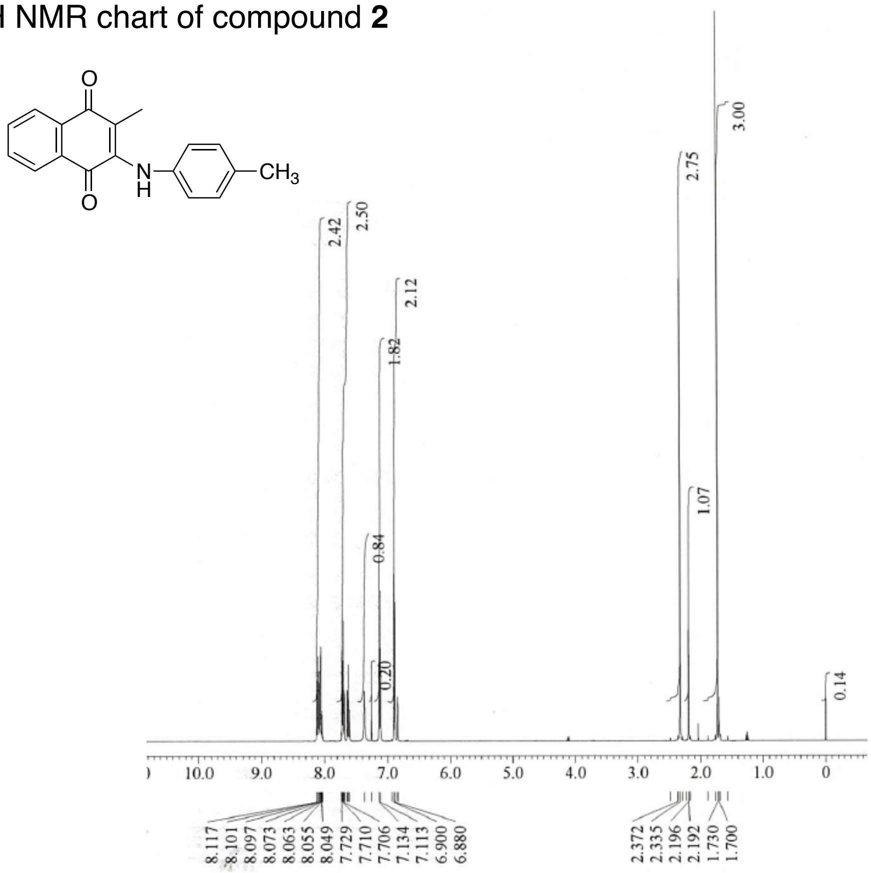

^{13}C NMR chart of compound **2**

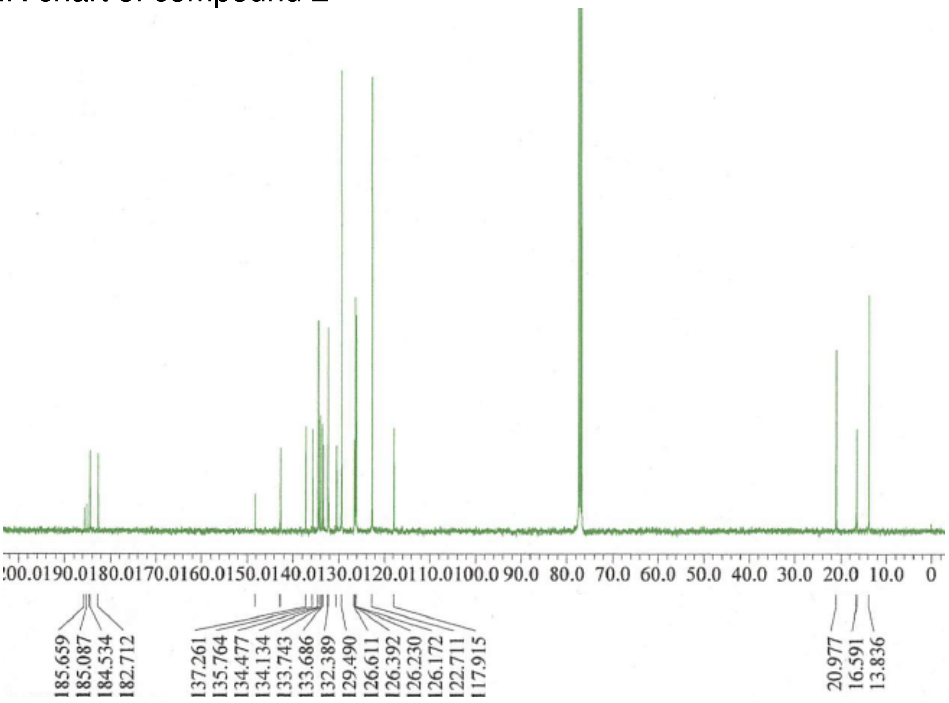

^{1}H NMR chart of compound **3**

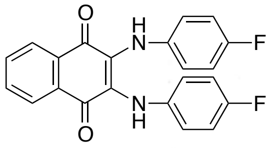

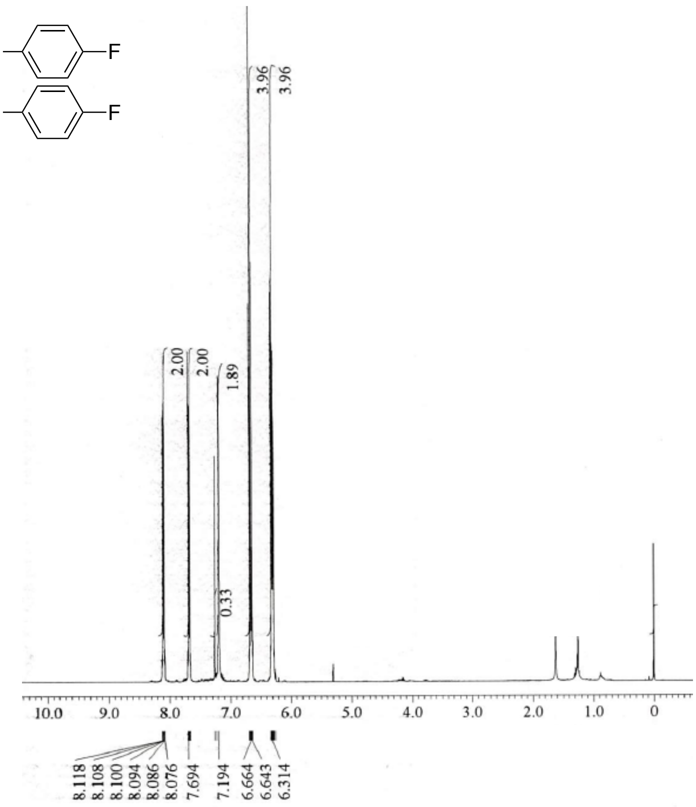

^{13}C NMR chart of compound **3**

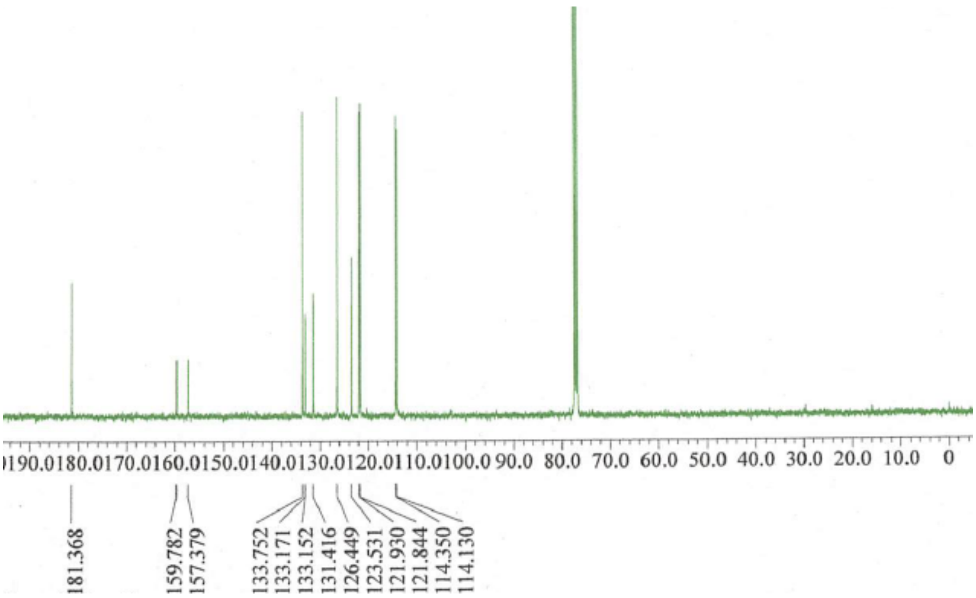

^{1}H NMR chart of compound **4**

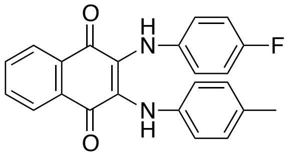

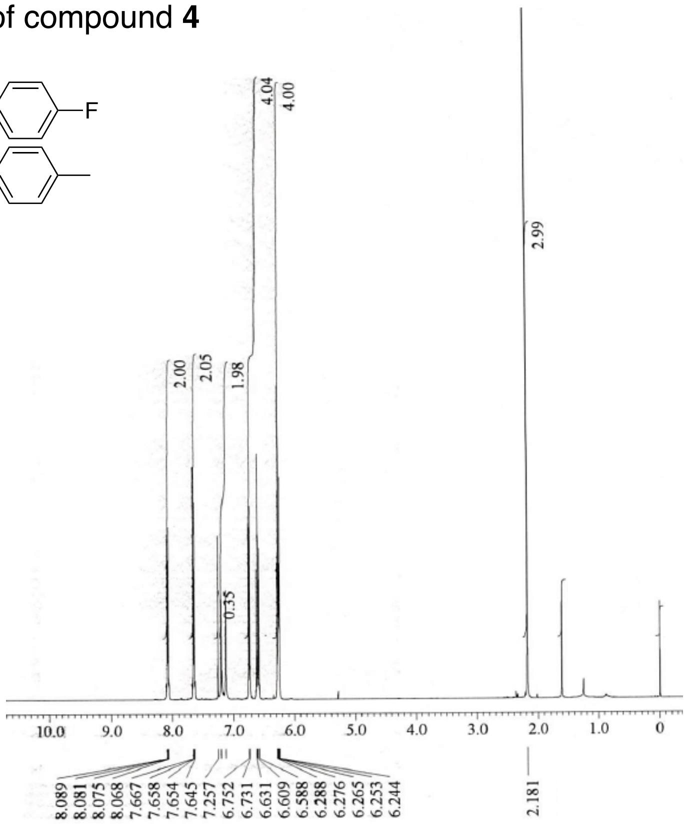

^{13}C NMR chart of compound **4**

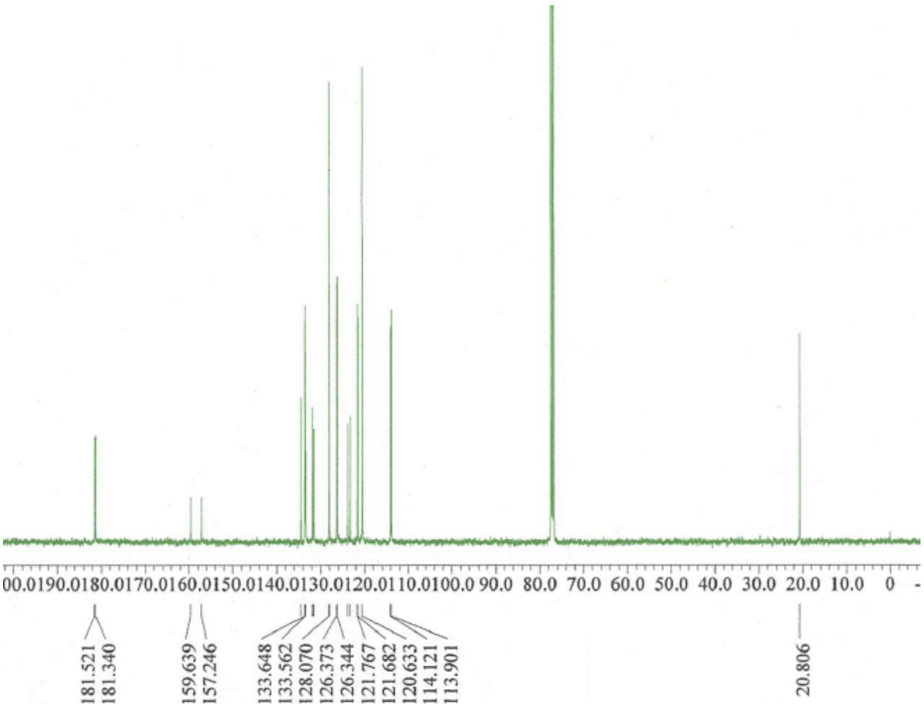

^{1}H NMR chart of compound **5**

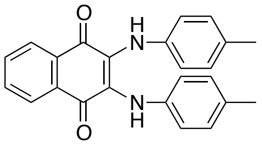

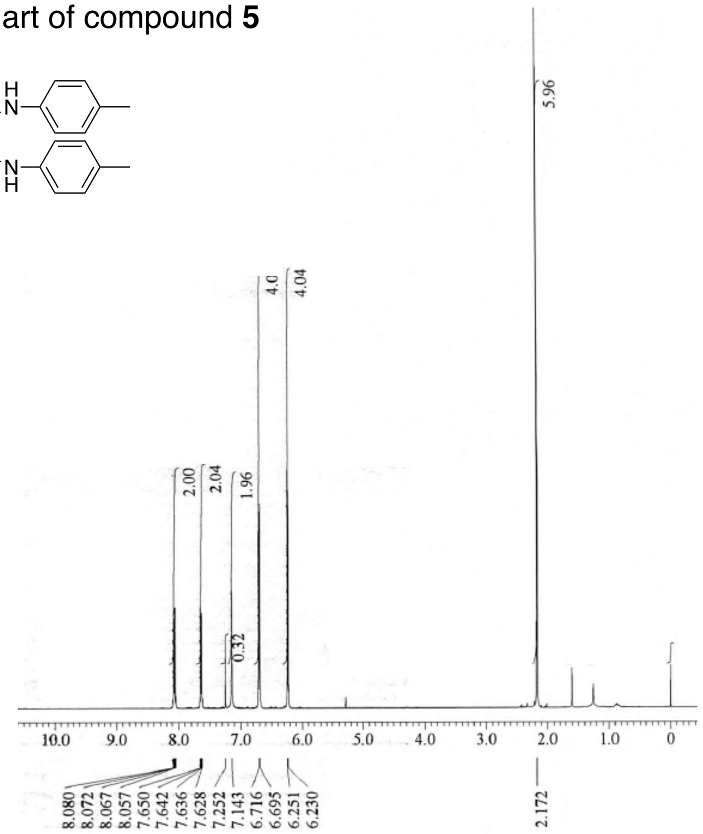

^{13}C NMR chart of compound **5**

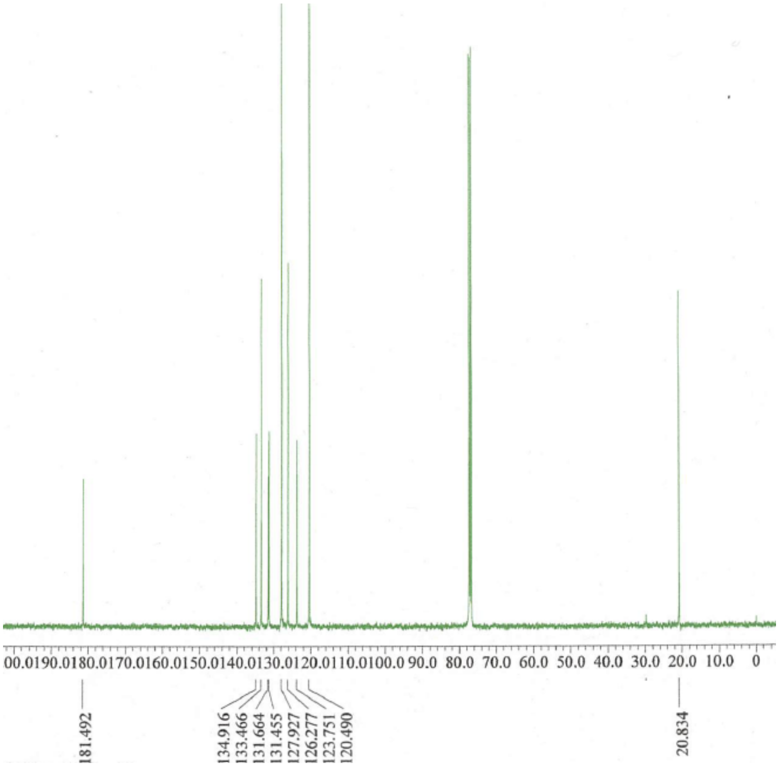

^{1}H NMR chart of compound **6**

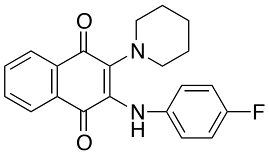

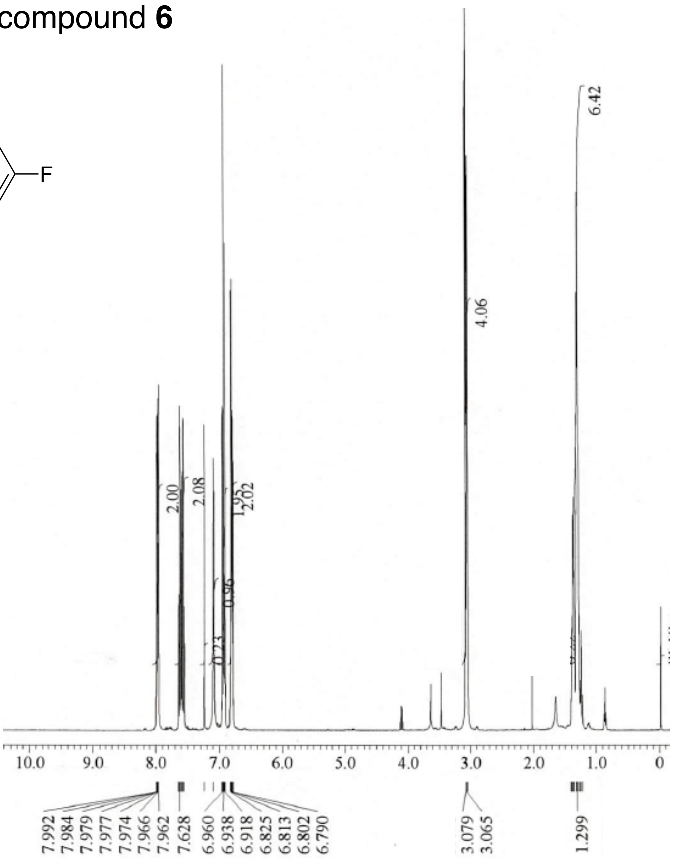

^{13}C NMR chart of compound **6**

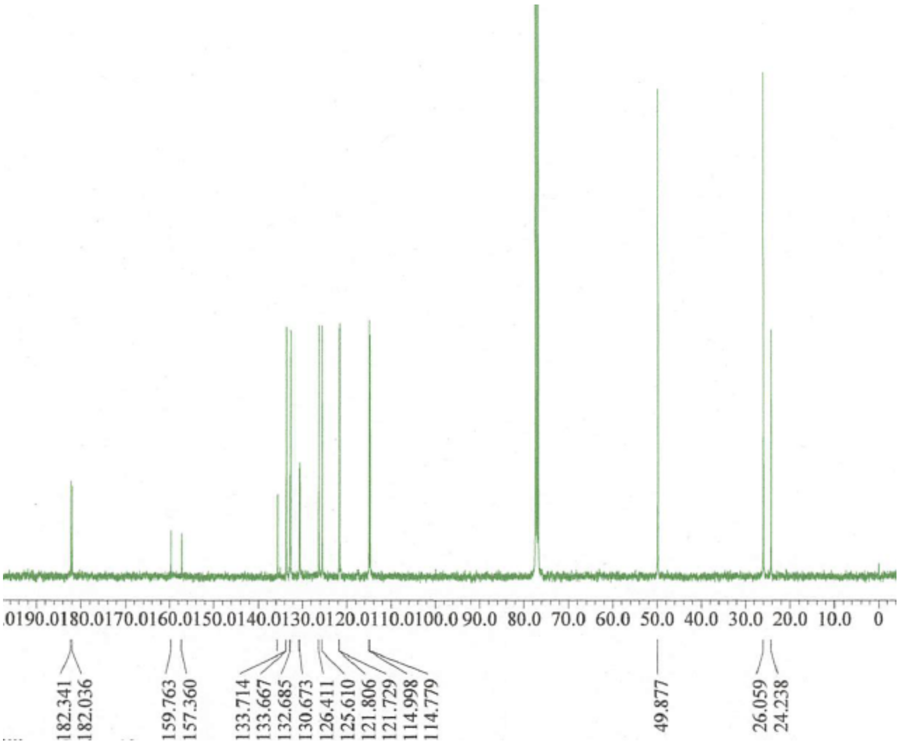

^{1}H NMR chart of compound **7**

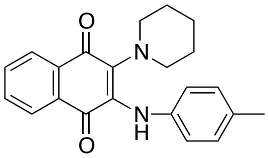

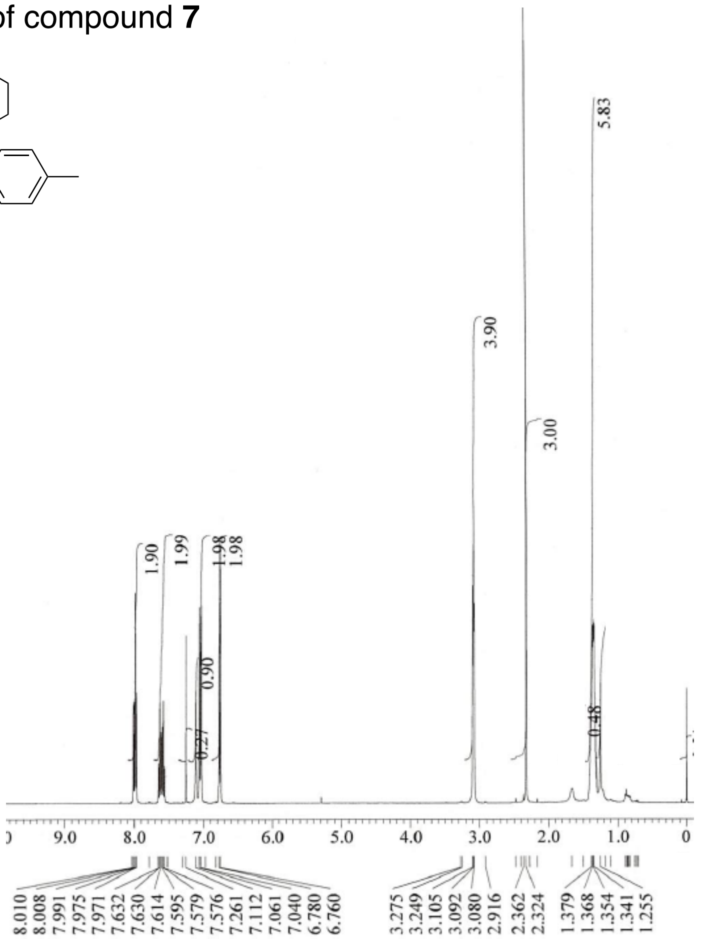

^{13}C NMR chart of compound **7**

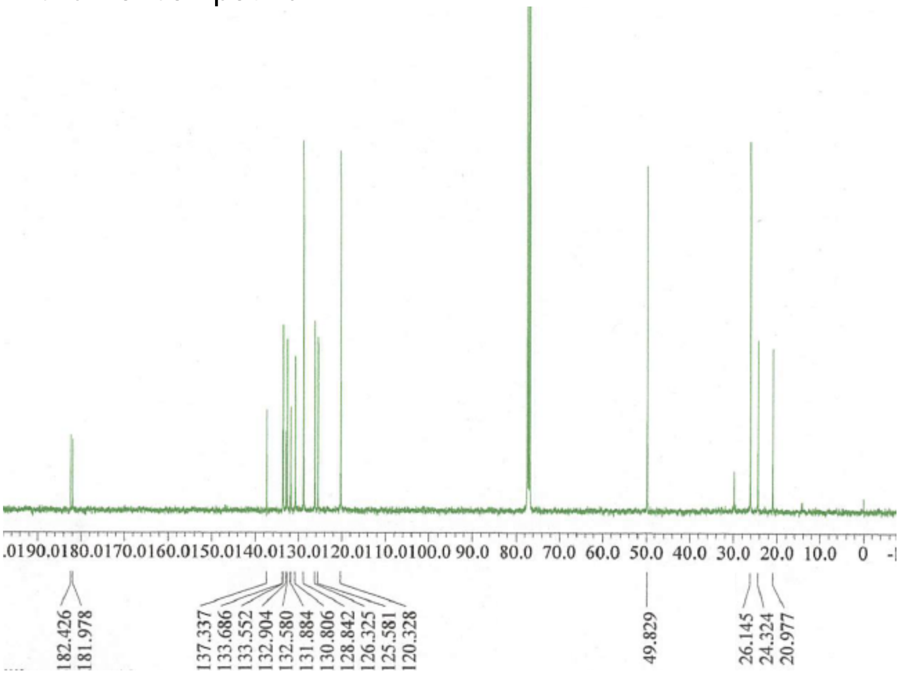

^{1}H NMR chart of compound **8**

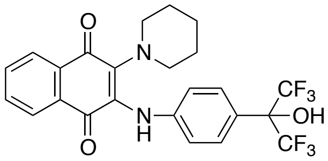

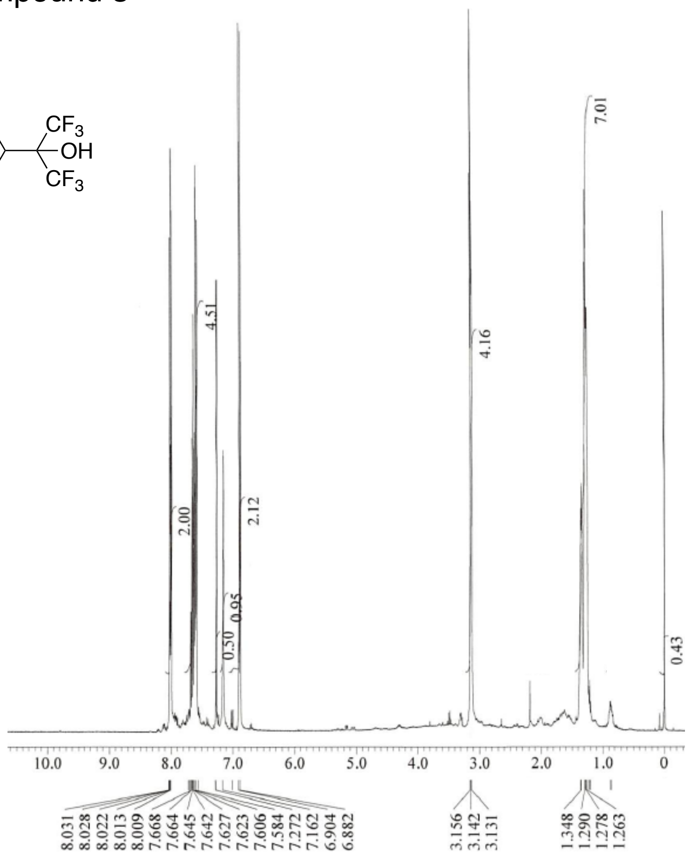

^{13}C NMR chart of compound **8**

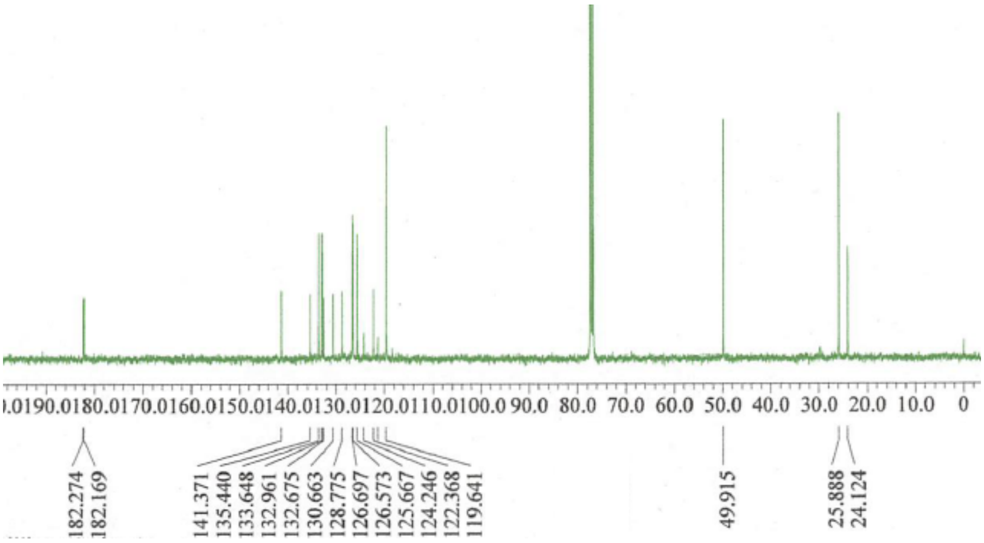

^{1}H NMR chart of compound **9**

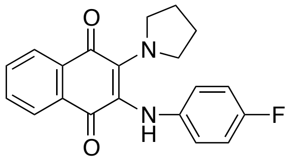

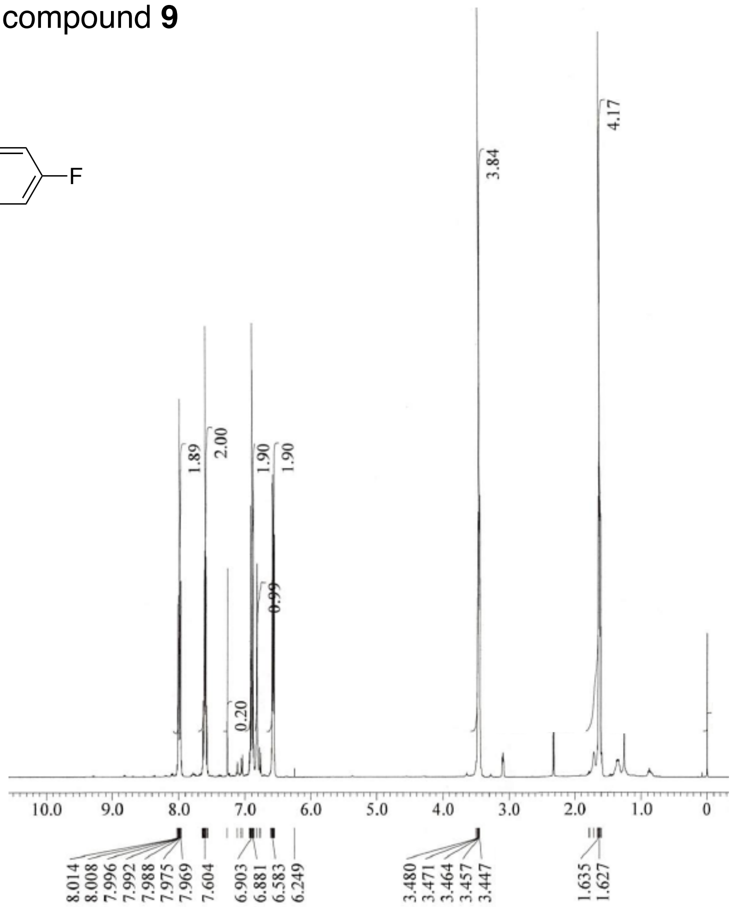

^{13}C NMR chart of compound **9**

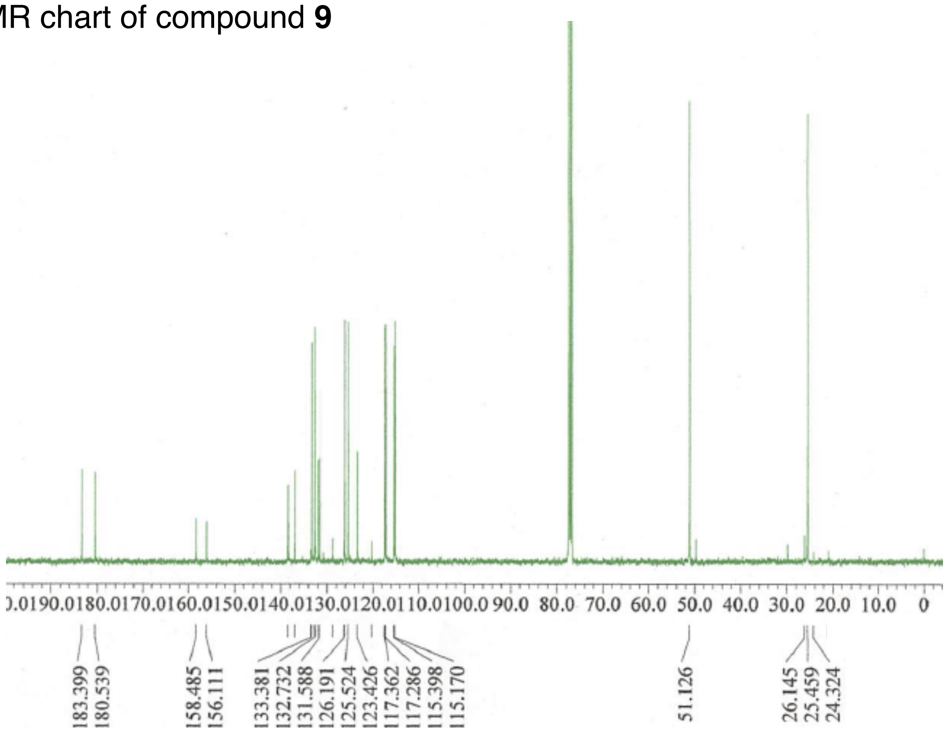

¹H NMR chart of compound **10**

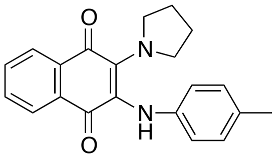

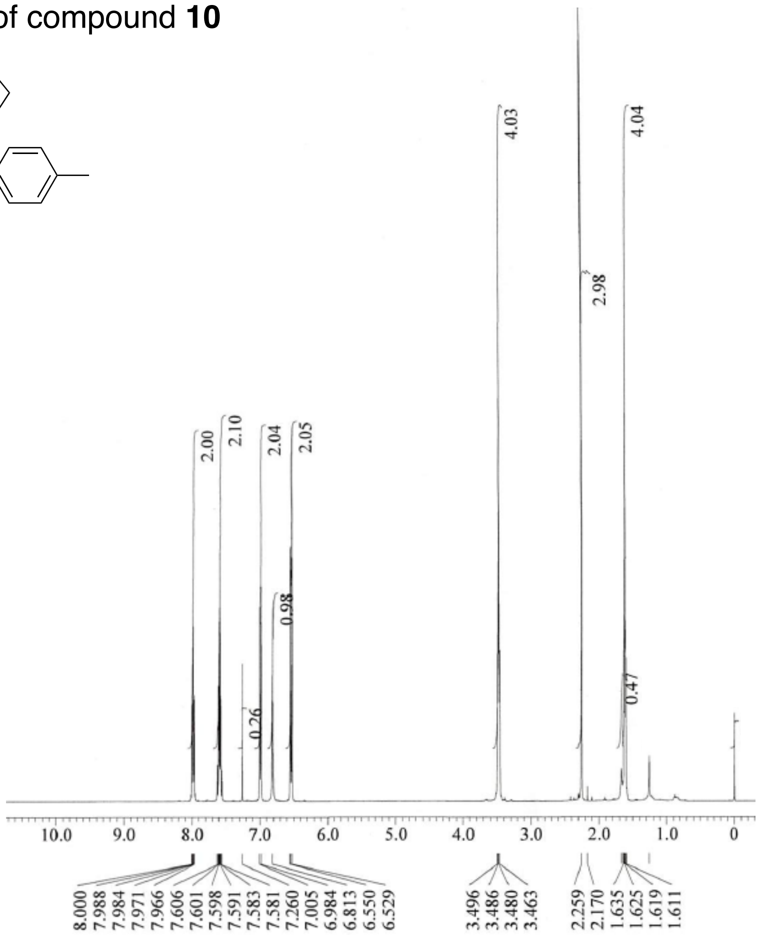

¹³C NMR chart of compound **10**

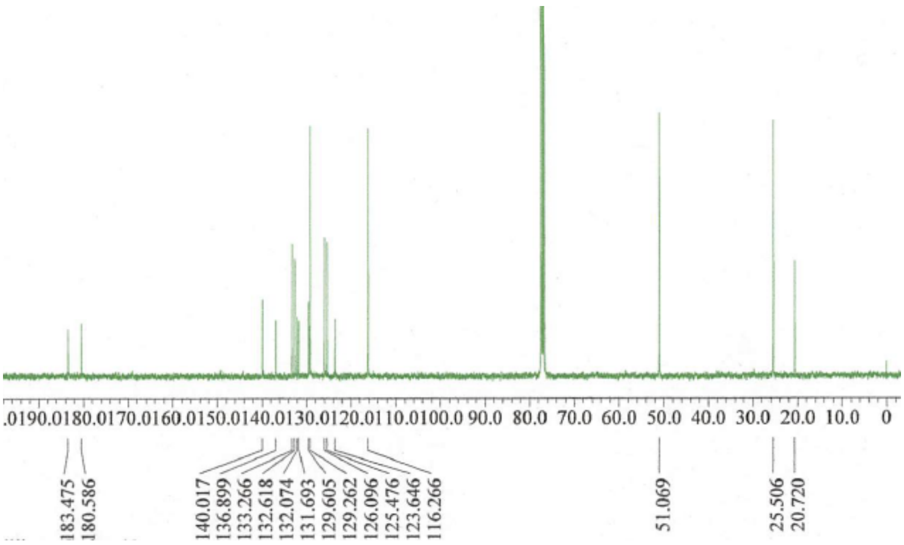

^{1}H NMR chart of compound **11**

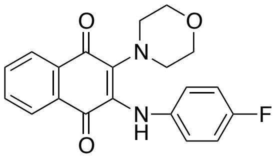

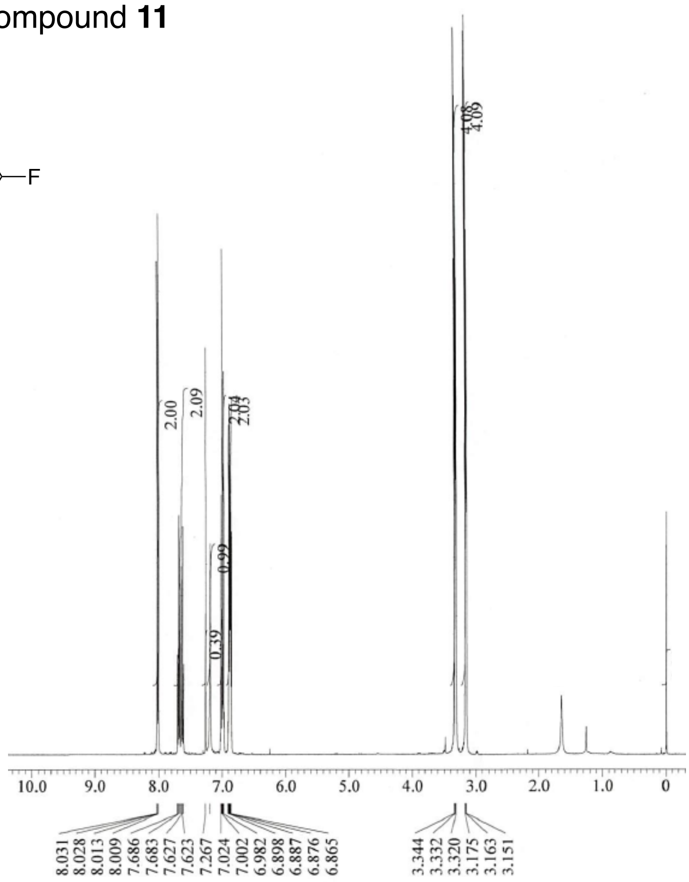

^{13}C NMR chart of compound **11**

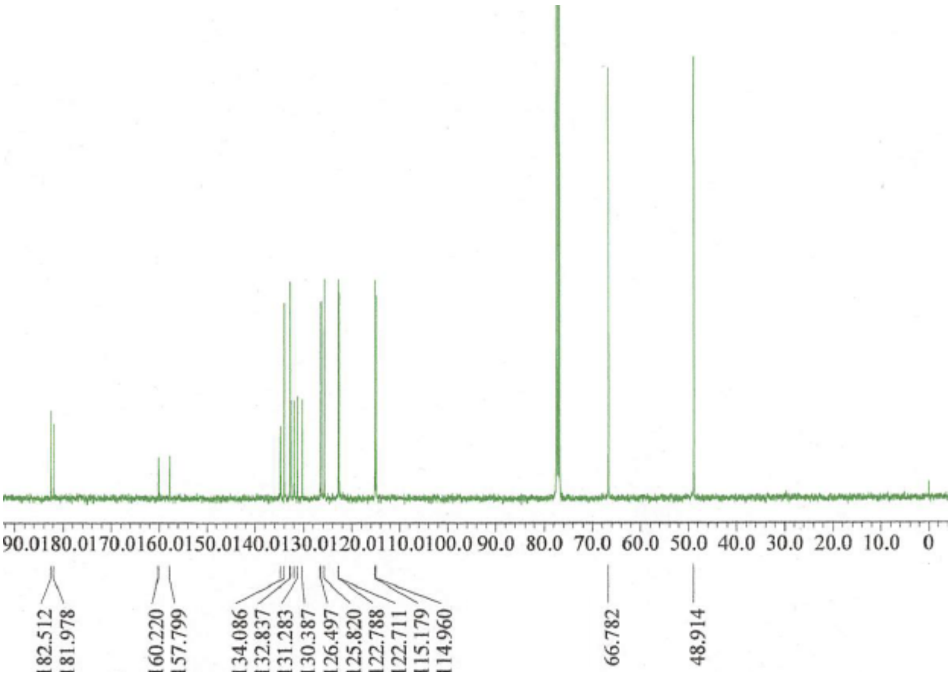

^{1}H NMR chart of compound **12**

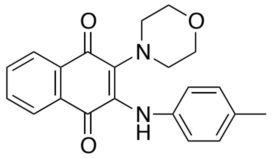

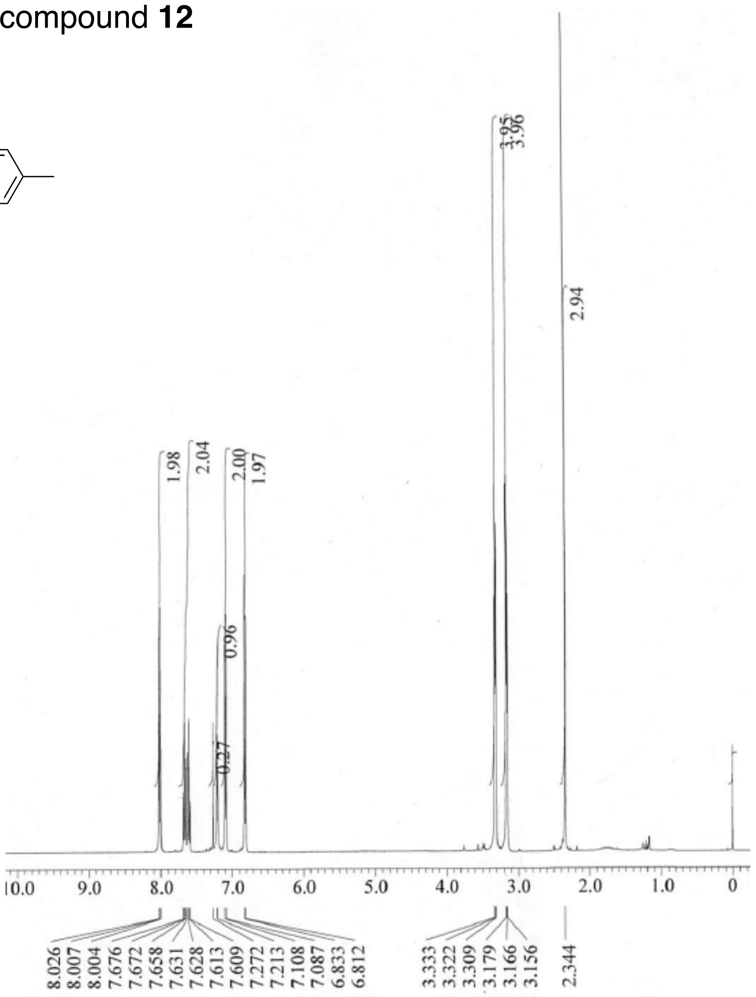

^{13}C NMR chart of compound **12**

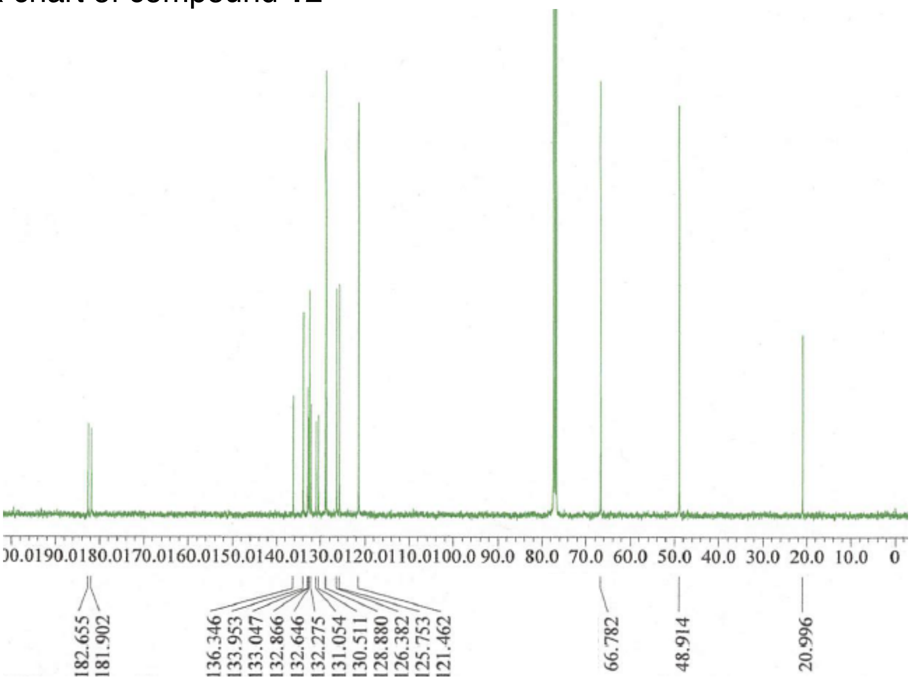

^{1}H NMR chart of compound **13**

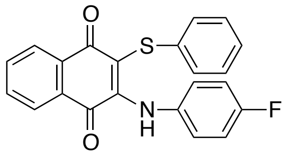

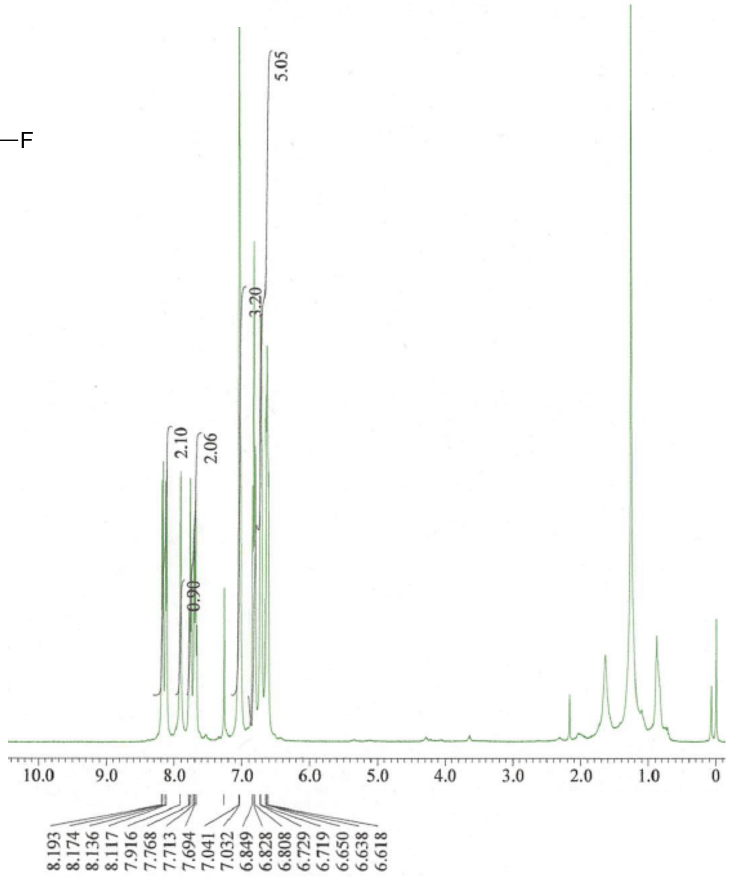

^{13}C NMR chart of compound **13**

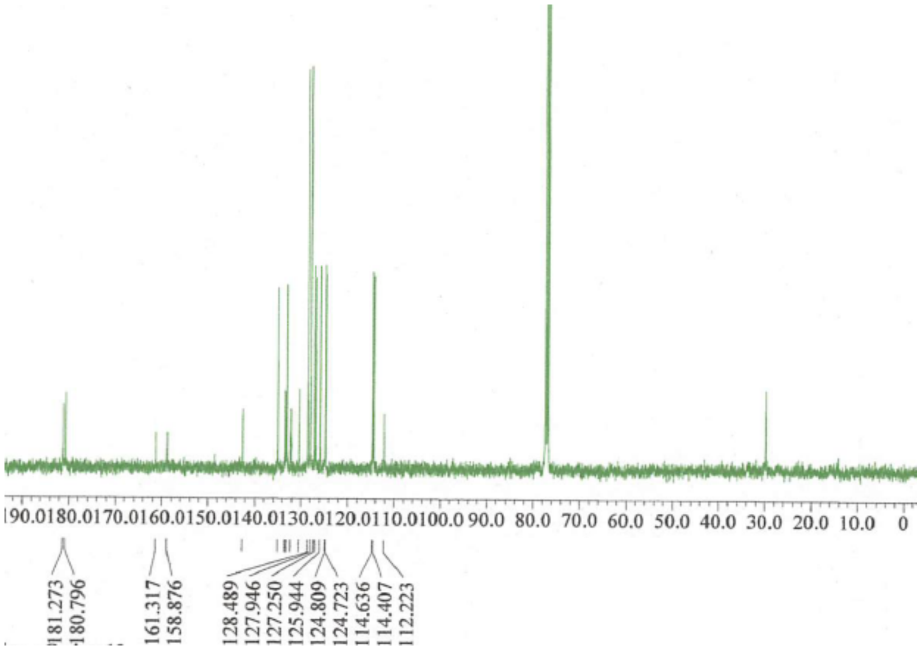

^{1}H NMR chart of compound **14**

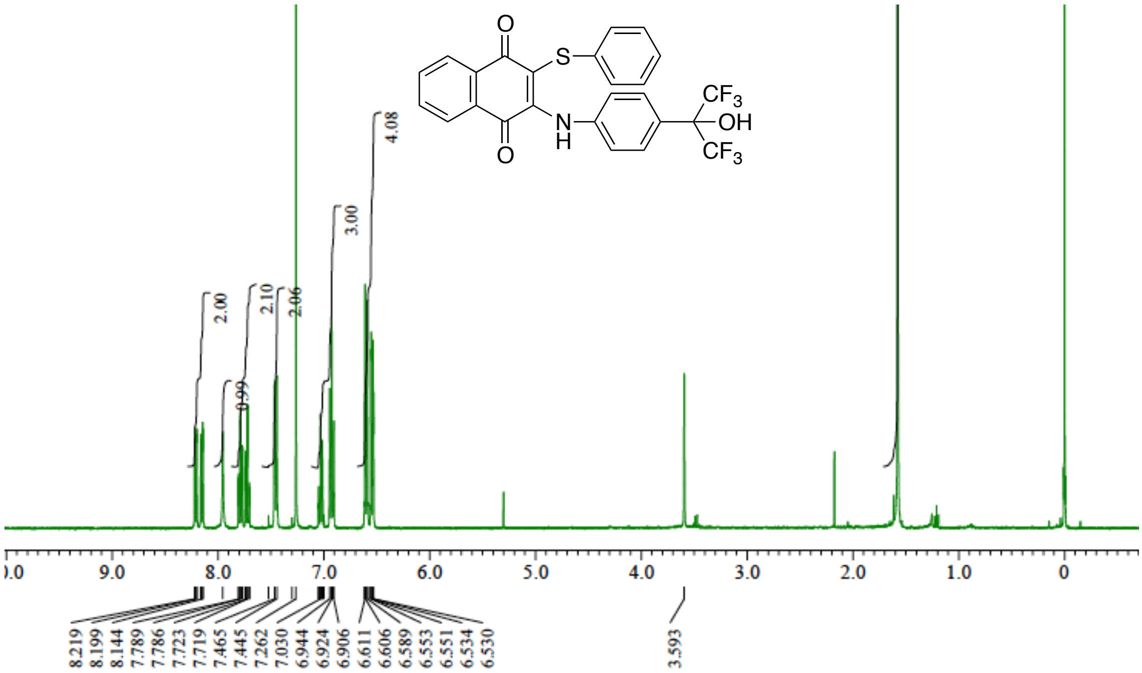

^{13}C NMR chart of compound **14**

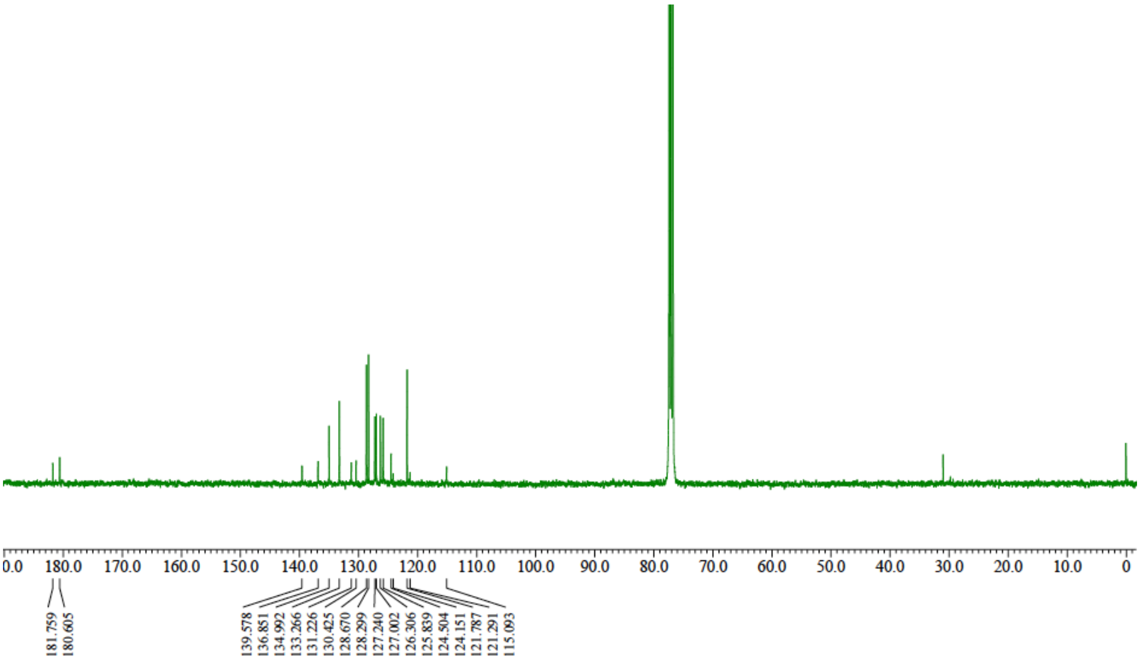

II. NMR data for compounds **1-5, 9, 10**

Data for compound **1**: ^{1}H NMR (400 MHz, $CDCl_3$): δ 1.72 (3H, s), 6.98-7.05 (4H, m), 7.33 (1H, br s), 7.64-7.72 (2H, m), 8.06-8.12 (2H, m); ^{13}C NMR (100 MHz, $CDCl_3$): δ 13.8, 115.7, 115.9, 118.3, 124.2, 124.3, 126.2, 126.3, 132.4, 134.4, 142.8, 158.4, 160.9, 182.5, 184.5; HRMS (M+H$^+$) m/z calcd for $C_{17}H_{13}O_2NF$ 282.0930. Found 282.0925.

Data for compound **2**: ^{1}H NMR (400MHz, $CDCl_3$): δ 1.73 (3H, s), 2.37 (3H, s), 6.88-6.90 (2H, d, *J*= 8.4Hz), 7.11-7.13 (2H, d, *J*= 8.4 Hz), 7.37 (1H, br s), 7.60-7.72 (2H, m), 8.04-8.11 (2H, m); ^{13}C NMR (100 MHz, $CDCl_3$): δ 13.7, 20.8, 117.8, 122.7, 126.2, 126.4, 129.5, 132.4, 133.4, 133.7, 134.1, 134.5, 135.8, 137.3, 142.8, 182.6, 184.4; HRMS (M+H$^+$) m/z calcd for $C_{18}H_{16}O_2N$ 278.1181. Found 278.1177.

Data for compound **3**: ^{1}H NMR (400MHz, $CDCl_3$): δ 6.28-6.31 (4H, m), 6.64-6.66 (4H, m), 7.19 (2H, br s), 7.66-7.69 (2H, m), 8.08-8.11 (2H, m); ^{13}C NMR (100MHz, $CDCl_3$): δ 114.1, 114.4, 121.8, 121.9, 123.5, 126.4, 131.4, 133.2, 133.8, 157.4, 159.8, 181.4; HRMS (M+H$^+$) m/z calcd for $C_{22}H_{15}O_2N_2F_2$ 377.1102. Found 377.1102.

Data for Compound **4**: ^{1}H NMR (400MHz, $CDCl_3$): δ 2.18 (3H, s), 6.24-6.28 (4H, m), 6.59-6.63 (2H, m), 6.73-6.75 (2H, m), 7.12 (1H, br s), 7.18 (1H, br s), 7.65-7.67 (2H, m), 8.07-8.09 (2H, m); ^{13}C NMR (100MHz, $CDCl_3$): δ 20.8, 113.9, 114.1, 120.6 121.7, 121.8, 123.3, 123.9, 126.34, 126,37, 127.9, 128.0, 131.5, 131.6, 131.8, 133.5, 133.6, 134,6, 157.2, 159.6, 181.3, 181.5; HRMS (M+H$^+$) m/z calcd for $C_{23}H_{18}O_2N_2F$ 373.1352. Found 373.1352.

Data for compound **5**: ^{1}H NMR (400MHz, $CDCl_3$): δ 2.17 (6H, s), 6.23-6.25 (4H, m), 6.70-6.72 (4H, m), 7.14 (2H, br s), 7.63-7.65 (2H, m), 8.06-8.08 (2H, m); ^{13}C NMR (100MHz, $CDCl_3$): δ 20.8, 120.5, 123.8, 126.3, 127.9, 131.5, 131.7, 133.5, 134.9, 181.5; HRMS (M+H$^+$) m/z calcd for $C_{24}H_{21}O_2N_2$ 369.1603. Found 369.1603.

Data for compound **9**: ^{1}H NMR (400MHz, $CDCl_3$): δ 1.62-1.64 (4H, m), 3.45-3.48 (4H, m), 6.56-6.60 (2H, m), 6.88-6.90 (3H, m), 7.58-7.62 (2H, m), 7.97-8.01 (2H, m); ^{13}C NMR (100MHz, $CDCl_3$): δ 25.4, 51.1, 115.2, 115.4, 117.3, 117.4, 123.4, 125.5, 126.2,

131.6, 132.0, 132.7, 133.4, 137.0, 138.4, 156.1, 158.5, 180.5, 183.4; HRMS ($M+H^+$) m/z calcd for $C_{20}H_{18}O_2N_2F$ 337.1352. Found 337.1351.

Data for compound **10**: ^{1}H NMR (400MHz, $CDCl_3$): δ 1.61-1.64 (4H, m), 2.26 (3H, s), 3.46-3.50 (4H, m), 6.53-6.55 (2H, m), 6.81 (1H, s), 6.98-7.00 (2H, m), 7.58-7.61 (2H, m), 7.97-8.00 (2H, m); ^{13}C NMR (100MHz, $CDCl_3$): δ 20.7, 25.5, 51.1, 116.3, 123.6, 125.5, 126.1, 129.3, 129.6, 131.7, 132.1, 132.6, 133.3, 136.9, 140.0, 180.6, 183.5; HRMS ($M+H^+$) m/z calcd for $C_{21}H_{21}O_2N_2$ 333.1603. Found 333.1597.
